# ConverSense: An Automated Approach to Assess Patient-Provider Interactions using Social Signals

**DOI:** 10.1145/3613904.3641998

**Published:** 2024-05-11

**Authors:** Manas Satish Bedmutha, Anuujin Tsedenbal, Kelly Tobar, Sarah Borsotto, Kimberly R. Sladek, Deepansha Singh, Reggie Casanova-Perez, Emily Bascom, Brian Wood, Janice Sabin, Wanda Pratt, Andrea Hartzler, Nadir Weibel

**Affiliations:** UC San Diego, La Jolla, CA, United States; UC San Diego, La Jolla, CA, United States; UC San Diego, La Jolla, CA, United States; UC San Diego, La Jolla, CA, United States; UC San Diego, La Jolla, CA, United States; UC San Diego, La Jolla, CA, United States; University of Washington, Seattle, WA, United States; University of Washington, Seattle, WA, United States; University of Washington, Seattle, WA, United States; University of Washington, Seattle, WA, United States; University of Washington, Seattle, WA, United States; University of Washington, Seattle, WA, United States; UC San Diego, La Jolla, CA, United States

**Keywords:** social signals, interactions, healthcare, patient-provider communication

## Abstract

Patient-provider communication influences patient health outcomes, and analyzing such communication could help providers identify opportunities for improvement, leading to better care. Interpersonal communication can be assessed through “social-signals” expressed in non-verbal, vocal behaviors like interruptions, turn-taking, and pitch. To automate this assessment, we introduce a machine-learning pipeline that ingests audio-streams of conversations and tracks the magnitude of four social-signals: dominance, interactivity, engagement, and warmth. This pipeline is embedded into ConverSense, a web-application for providers to visualize their communication patterns, both within and across visits. Our user study with 5 clinicians and 10 patient visits demonstrates ConverSense’s potential to provide feedback on communication challenges, as well as the need for this feedback to be contextualized within the specific underlying visit and patient interaction. Through this novel approach that uses data-driven self-reflection, ConverSense can help providers improve their communication with patients to deliver improved quality of care.

## 1 INTRODUCTION

Primary care providers’ communication [4] with patients influences patient care and health outcomes. Good communication skills on the provider’s side can improve patient understanding, trust, and clinician–patient agreement, thereby creating better self-care skills in patients [93]. On the other hand ineffective patient-provider communication, sometimes driven by implicit racial biases towards patients from historically marginalized groups [26], contributes to adverse outcomes [3, 61]. Despite the importance of effective communication skills, training interventions for providers are limited and often lack specific feedback tied to recent patient encounters [21].

With advances in conversational analysis, technology could help to address these concerns by automatically assessing the quality of providers’ communications and help reduce the impact of potential implicit biases [7, 41] on patient care. Specifically, technology interventions could identify communication behavior breakdowns that occur when healthcare providers interact with patients by investigating the *social signals* associated with the interaction [71, 96]. Social signals are defined as the complex aggregates of behaviors that individuals express in their attitudes towards other speakers within the current social context. Since they also depend on the state of other speakers in the conversations, social signals go beyond an individual’s emotions or non-verbal behaviors [71].

The process of identifying social signals and tracking their state is referred to as Social Signal Processing (SSP) [96]. Based on relational communication theory [12, 15], one representation of social signals in clinical interactions between patients and providers is interpersonal affiliation (i.e., rapport, trust, warmth) and conversational control (i.e., dominance, influence, authority) [45]. Another widely used representation of communication behaviors for medical encounters is the Roter Interaction Analysis System (RIAS) [78], which encompasses twelve social signals. Social signals have also been used to model conversations outside of healthcare, especially around leadership traits detection [8], audience-presenter interactions [29], teacher-student interactions [37].

While RIAS is one of the most common systems to study social signals in medical encounters, its application to annotate patient visits is labor intensive. Annotation requires skilled and specially trained human observers to manually code each visit for each social signal. This requires annotation process storing large numbers of recorded visits, and spending many hours watching and analyzing those visits, which limits scalability and accessibility to the data.

In contrast to traditional manual assessment, automated SSP methods have shown promising results towards computationally assessing encounters based on non-verbal cues [1, 33, 51, 54]. Non-verbal behaviors, such as body language, turn-taking and interruptions have been used to describe specific social behaviors [1, 45]. Head movement and other body gestures are often indicators of dominance [8]. Measures of turn-dynamics [84] and speech [48] are also used to describe engagement for casual conversations.

SSP advances create opportunities to raise clinician awareness of communication patterns across different patients. Automatic assessment of clinical visits can ensure that providers receive feedback about the quality of communication without having to wait for manual assessment. Our recent work on understanding providers’ willingness and need for such feedback has reiterated the importance of receiving feedback in a timely manner [31, 77]. Furthermore, innovative training interventions that leverage SSP through automated sensing and feedback have the potential to more efficiently scale and to assess the quality of clinical communication at a wider level, without the need of manual annotations, or the integration of communication coaches, both approaches that are costly, hard to implement and overall not scalable.

### Contributions

While existing research includes efforts towards computationally sensing clinical interactions [33, 45, 57], and indicates how social signals are promising to understand social behavior [45], there has been limited effort to automatically extract and infer complex social signals that go beyond non-verbal cues from patient-provider interactions in near real-time. Current work also does not provide a complete system that integrates the RIAS social signal processing models into a clinician-facing feedback tool that provides effective interfaces for clinicians to understand social signals and their interactions with patients. Our work fills this gap contributing to the current research landscape in these distinct ways:

- We analyze social behaviors in 100+ primary-care clinical visits to identify social signals that can be *visualized and presented to clinicians* to impact patient-centered care
- We describe an *interpretable SSP approach* to computationally track changes in social behaviors during clinic visits from audio recordings; this approach allows clinicians to *directly map their interactions to social signals* and elicit actionable feedback
- We describe the *design of a web-based tool* that integrates our SSP pipeline and introduce *ConverSense*, a tool to assist providers in reflecting on their social signals
- We evaluate the *usability and user perceptions of the ConverSense tool* for providers and discuss insights for future development

Our explorations reveal that despite a high learning curve, clinicians find it quick and easy to understand the paradigm of social signals. We found that. providing communication feedback through visualizing social signals helps them uncover their communication patterns, but also that more knowledge of the underlying interactions is required to make the feedback actionable.

While our approach is general with respect of the media used (i.e., video or audio), and can be extended to multiple social signals during clinical encounters, the current work focuses on audio-based signals. Sound is the most ubiquitous modality for recording interactions, and arguably the least intrusive to capture in clinical practice.

## 2 RELATED WORK

Our work builds on and extends a number of related work in different areas. In this section, we outline previous research on patient-provider communication analysis, the application and use of social signals, and existing gaps in computationally sensing clinical interactions.

### 2.1 Patient-Provider Communication Analysis

Manual coding systems have been historically used to analyze patient-provider communication during medical interactions. Among the most widely used is the Roter Interaction Analysis System (RIAS) [78], which examines many facets of the patient-provider relationship based on social theory and exchange models. Rooted in social exchange theory [63], RIAS allows for investigating the dynamics of the patient-provider relationship through measures reflecting socio-emotional and task-focused elements of the medical exchange. In particular, the RIAS Global Affect Rating (GAR), measures the expression of the overall emotional dynamics of a clinical visit, and has been used extensively to study patient-centered communication. While RIAS holistically looks at an entire patient-visit, GAR scoring involves segmenting each interaction into the 3–5 minute long “slices,” which allows for a more localized analysis of the communication. This “thin slicing” creates the unique opportunity to assess socio-emotional behaviors over time within the local context [79, 89].

The use of analysis systems that code medical dialogue has been able to predict a variety of patient and provider outcomes associated with race, such as disparities in communication quality, information giving, shared decision making [87], as well as affective communication [92]. Due to such associations, patient-provider communication is often plays a central role in detecting biased interactions [7, 41] as well as identifying patient empowerment strategies [20, 31, 39]. Researchers have designed qualitative descriptors of frictions in patient-provider communication [14] and motivated the need to design technologies to ensure patients are treated with respect [14, 33, 86]. Despite the need to develop technologies is justified, there has been limited attention towards developing technology to characterize behaviors associated with bias in healthcare interactions.

Introducing novel approaches to improve patient-centered communication can improve the quality of care by giving providers feedback on communication behaviors associated with implicit bias [26] that can be used to improve patient satisfaction [62] and reduce healthcare disparities [34, 43, 67]. Despite their efficacy, frameworks like RIAS rely on human effort to code for these behaviors that can affect outcomes, which restricts their scalability to every provider and each visit. This creates a major opportunity to introduce computational tools that can help to automate the coding process through machine learning.

### 2.2 Social Signals and their Use in Clinical Interactions

Social signals allude to verbal and nonverbal communication behaviors, such as what is said and how it is said through body movement and placement, facial expression, or voice (e.g. talk time, interruptions, tone). As described by Vinciarelli et al. [96], social signals are “complex aggregates of behaviors that individuals express in their attitudes towards others.” While social signals encompass emotions and sentiments, they are far more nuanced due to the fact that they are not just a state of an individual’s mind but also depend on the state of the other speakers in the conversation.

In Entendre, Hartzler et al. [45] showed that social signals can be associated with nonverbal communication cues in clinical encounters. Through a literature review, the authors showed how these cues, including vocalics, such as interruptions, pauses, and speaker turns, map to social signals associated with patient-centered care, namely control and affiliation.

Various examples show how social signals affect clinical interactions. For instance, Henry et al. [46] found that increased listening and warmth during patient-provider interactions led to greater patient satisfaction. Changing non-verbal behaviors can also influence perceived warmth of providers [53]. Street and Buller [91] showed that patient-satisfaction, provider-engagement, and emotional-caring are related. Yet, research also showed that implicit attitudes can affect communication and negatively impact patients’ experiences of their medical encounters [26]. Despite this rich literature, no computational model exists that is able to link raw audio features and associate them with these social signals in the context of healthcare.

### 2.3 Computationally Sensing Clinical Interactions

Social Signal Processing (SSP) is a computational approach that analyzes and synthesizes social signals observed in interpersonal interactions [16]. Formative work on *Honest Signals* [71] has shown that social signals like activity, consistency, influence, and mimicry could be sensed from a person’s social interactions. Specifically, in the clinical context, SSP has been used to assess communication and guide feedback tools that encourage empathetic, patient-centered communication [33, 45, 57, 58, 98]. The rationale for these efforts stems from the fact that verbal and nonverbal communication skills can be measured for quality [25] and these measures allow for improvement of skills [88].

The EQ-Clinic study [57, 58] used tele-health systems to show feedback for medical students. It captured information about verbal and non-verbal cues in the conversation to guide student practitioners to interact better with patients. It was found that speaker-turns, smile intensity, frown intensity, and head nodding were powerful features. The ReflectLive tool [33] was designed for understanding patient-provider interactions and presenting them feedback in real-time. The system took audio and video information from telehealth calls and described the conversations with audio, body movement, and eye-gaze patterns. It showed the use and importance of interruptions in conversations. However, the system only showed metrics based on multimedia data, and the insights drawn from the conversations were limited to those metrics without any context.

While most of these systems show some measure about the conversation, they do not take into account the socio-emotional context of the interaction and do not model those relational communication metrics directly into social signals. As described in formative studies by Bascom et al. [4], Dirks et al. [31], purely showing audio or video-based raw indicators (e.g., number of pauses, time looking at the patient, etc.) may not be informative for providers looking to improve their conversations with patients.

More extensive work in trying to understand the role of the interpersonal context was introduced by Hartzler et al. [45]. Their Entendre study showed that deeper contextual information about the quality of clinical interactions can be shown through social signals expressed by speakers during social interactions [96]. This work proposed the use of affect ratings to score conversations, however, the work was limited to a Wizard of Oz study, and a functional system was not implemented.

Although much progress has been made toward computationally sensing clinical interactions, no research so far has introduced a social signal processing model for automatically assessing patient-provider communication that takes into account the socio-emotional quality of the speakers and can show metrics beyond non-verbal cues like speakers’ turn-taking and number of interruptions. To address this gap, we investigate AI-generated social signals to characterize and provide feedback to providers on their communication with patients.

## 3 SOCIAL SIGNAL PROCESSING PIPELINE

This section discusses the process of introducing a novel Social Signal Processing (SSP) pipeline to translate nonverbal cues detected in audio recordings to social signals in patient-provider interactions during clinical visits. We describe our design considerations, the creation of a specific dataset, and the development and evaluation of a machine learning based pipeline to extract social signals.

### 3.1 Requirements

To create an effective and trustworthy pipeline we need to ensure that any sequence of information processing modules we build can provide an interpretation of the clinical conversation. Since we aim to design a pipeline to infer a qualitative measure of social behavior, we start by defining specific factors that need to be considered in the design process.

#### Near Real-time System —

While previous work attempted to show communication feedback to providers in real-time [33, 45, 58], Dirks et al. [31] and Bascom et al. [4] have shown how this is disruptive and how providers valued contextual reflective feedback that is available *soon after the visit*. To address the intrusiveness of real-time feedback, but still satisfy the need of post-visit feedback, we focus on a *near real-time* modality that is not impacting the visit itself (does not show any of the results during the visits), but is available right after the visit is completed. Our proposed approach is therefore based on a system that would have to function in near real-time at the minimum to impart feedback to providers right after the visit is over. This implies that the machine learning pipeline has to complete the processing within minutes of the completion of a visit.

#### Interpretable & Evidence-driven —

Recent studies have found that providers’ or patients’ mistrust in AI predictions can translate into damaging provider-patient relationships [56] and that providers’ trust in clinical decision support systems is very strongly dependent on models rooted in background evidence [13, 17]. To ensure background evidence, our machine learning features should be conceptualized based on known associations in literature about clinical communication. In addition to improving trust, an interpretable model also helps providers get specific and actionable feedback on their communication.

#### Choice of recording modality —

Our choice of modality used for Social Signal Processing is driven largely by practical observations. It is known that participants’ behavior is often altered when being recorded [55]. In prior clinical research, patients who had already consented to being recorded also marked discomfort in communicating in the presence of a recording system [38]. It is however, also known that recording audio can be less intrusive than video [99]. Furthermore, setting up audio recording devices in clinics is much easier than video cameras, and can accommodate for a range of placement positions as compared to video. Finally, for many vocalic features, the literature already demonstrated a strong relationship with social signals [45]. For all these reasons, we believe that primarily focusing on building an audio-driven social signal processing pipeline is a promising initial approach.

### 3.2 Data Preparation

We created our own dataset annotated with the RIAS guidelines derived from medical communication studies. Under RIAS, human annotators (or manual coders) analyzed each interaction post-hoc and scored each behavior (e.g. dominance, interestedness, etc.) on a Likert scale from 1 to 6 for small successive slices (generally 3–5 minutes) of the interaction [79]. In that rating, 3 is treated as neutral or average behavior and scores below and above three indicate a deviation from the normal behavior. RIAS is designed to identify anomalous behaviors and assist the coders by first comparing it to a globally acceptable “neutral” behavior (scored as “3”) and then scoring the deviations from such a baseline. For example, a provider or patient can show disinterest or increased interest in different parts of the same conversation which can be coded by a value of less than “3” or greater than “3”. Based on Roter et al. [79], we defined a slice as a subset of an interaction, lasting three minutes. We coded each interaction-slice using an in-house data-annotation tool designed for this task.

#### Dataset —

We used, with permission, the *Establishing Focus* (EF) dataset [62], which is a corpus of clinical recordings of interactions between patients and primary care providers. These interactions are primary outpatient visits where both speakers are talking in a stationary position. In the corpus of data, the majority of the patients were female (56.6 percent), middle-aged (44.5 percent ages 40–60), and White American (74.8 percent). We sampled 10 providers with and their recorded visits with different patients for analysis, resulting in a total of 103 patient-provider interactions that have a duration of mean = 34.38, std = 15.78 minutes. Dividing these interactions into successive blocks of three minutes results in 531 slices of usable interaction.

#### Manual Coding —

As required by RIAS guidelines, all annotators were first trained to code for a pre-defined example set, until agreement was reached and an Inter-Rater Reliability of 0.7 was achieved. Since the audio recordings in our dataset were collected in a different location and a long time ago, none of the annotators had any personal connection or relations to the speakers in these recordings, effectively reducing annotation bias in the coding. Our first pass on the EF data generated 80.7% agreement between the coders. Slides with disagreements were discussed by the coders in guidance with communication analysis experts to achieve complete agreement on the labels.

#### Labels —

From our coding we observed that the labels from 1 to 6 were very sparsely distributed. To address this imbalance in our data, yet still derive meaningful insights, we abstracted the system and modelled the task as a prediction of three classes – **below-baseline (1–2)**, **baseline/neutral (3)** and **above-baseline (4–6)**. While we acknowledge that this is a limitation, and that this will lead to the inability to identify exact intensities of these social signals, we believe that understanding deviations from baseline can still provide significant insights into provider-behavior.

### 3.3 Social Signal Identification

For our work we focus only on signals with positive affect in RIAS —Dominance/Assertiveness, Interest/Attentiveness, Friendliness/Warmth, Responsiveness/Engagement, Sympathetic/Empathetic, Respectfulness, Hurried/Rushed, Interactive/Fully Involved. To better outline our identification process, we describe below how we have considered signals that have known associations with vocalic non-verbal behaviors in clinical settings, signals with no evidence of direct impact to clinical care, and signals that have been used beyond the healthcare domain.

#### Impactful social signals in patient-provider interactions —

To understand which of these signals to use, we looked at their association with patient-provider communication. Specifically, among the social signals described in RIAS, Hartzler et al. [45] found associations between vocalic descriptors for dominance and warmth. We learned that higher pause and interruption counts, higher intensities, and higher provider talk-times are associated with a higher provider-dominance. We also learned that a less vocally active provider, lower variation in pitch and high response latency are all factors associated with the perception of warmth [45]. Other work pointed to *interactive* conversations as exchanges where both speakers contribute towards speech in a given time frame, which signals affiliation. Interactional synchrony also has positive associations with outcomes [60, 91], while interactivity is closely related with overall talk-time, and high interactivity is marked by more turns, briefer duration of turns and lower number of pauses [80]. Finally, Roter et al. [79] and Hsiao et al. [48] showed that engagement is often related to healthy turn-taking among the speakers and faster response times between turns; thus, turn-taking and pauses may be effective feature concepts for study engagement.

#### RIAS signals with no evidence of clinical care impact –

While this existing work from above allows us to successfully outline a number of links between social signals and clinical outcomes for four RIAS social signals — Dominance, Interactiveness, Warmth, and Engagement — our literature research did not find significant linkage with clinical outcomes for the other RIAS social signals that can be extracted using audio features. In particular, while *interest* would influence clinical interactions, extracting measures of interest will need to be based on the content of the conversation, something that our models explicitly avoid by design. Also, we could not find prior research that links *sympathetic/empathetic* social signals to vocal behavior during clinical interactions.

#### Modeling identified signals beyond health –

The choice of the four signals above is further reinforced by previous work outside of patient-provider interactions that validates the choice of these four signals in addition to the clinical context: *Dominance* has been studied often as an overall personality-metric [49, 50]; *interactiveness* and *engagement* have been studied in the context of audience-presenter interactions [29] and teacher-student interactions [37]; warmth has also been studied as a metric for professional behavior in organizations [28] and improving personalization of robots [72].

Based on the considerations from a clinical point of view, and the validation of the social signals at a more general level, we feel confident in designing our machine learning pipeline based on predicting these four RIAS social signals: **dominance, interactiveness, engagement** and **warmth**. Table 1 shows the summary of our dataset, and the final labels after clustering and signal selection.

### 3.4 Feature Extraction

From our review above, we identified two major kinds of audio features critical for modeling social signals, namely *turn-based* features and *speech-based* features. To look for either, we first need to identify *who spoke when*, a task commonly referred to as *speech diarization*. We use *diart* [27], a recent open-sourced real-time speaker diarization algorithm, to detect turn-taking. Turn-based features look at the dynamics of turn-taking between the speakers and present a meta-view of the conversation; speech-features provide more subtle descriptions of behavior, based on the utterances extracted from the dialogues. Social signals are measures of an interaction and hence it is important to have features describing both the provider and the patient to model the social signal levels for either speakers. Since our labels are for slices of a few minutes, our features are also designed per slice.

Measuring statistics for individual utterances often leads to inconsistent information since shorter turns tend to have limited variation in both, the temporal and frequency domains. Similar to Borrie et al. [10], we append all speaker utterances per speaker, within a given short context (slice) to get large enough speech signals to draw features from. The compilation of speech utterances for each speaker in a given slice can then be used to extract different features that have shown promise for other similar tasks. This preprocessing step creates a single utterance for each speaker, the patient and the provider. From the two compiled speech segments, we identify popular descriptors of speech inspired from recent work on conversational analysis using speech across domains, such as conversational entrainment [9, 10], emotion recognition [48, 97, 100] and laughter detection [95].

Using ideas from recent work discussed above, we extract slice-level features with known associations with non-verbal vocalic behaviors (e.g. loudness, pitch, etc.) as well as socio-emotional behaviors. Below is a summary of our feature set.

1. *Turn-Taking Behavior* — Measures different statistics describing turns taken between the provider and patient
2. *Interruptions* — While interruptions are more than simple conversation overlaps [66], for the sake of computation, we mark every instance of turns overlapping as an interruption. We define statistics around interruptions as well as the turn length after an interruption [50]
3. *Pauses* — When there is a gap between the end of a turn and the start of the next turn, we treat it like a pause in the conversation. We derive various statistical features based on the duration and occurrences of pauses
4. *Mel Frequency Cepstral Coefficients* — Represent the short-term power spectrum of a frame. The lower order MFCCs correspond to the frequency response of the vocal tract, while the higher order MFCCs respresent responses to the source signal. We use the first 13 cepstral coefficients
5. *Spectral Centroid* — Tracks the mean of an (assumed) Gaussian distribution of the normalized energy across frequencies and acts as a central measure of voice
6. *Spectral Rolloff* — Measures the frequency range which contains over 85% of the max energy; it acts as a measure of variation in a given frame
7. *Pitch* — Represents the base peak frequency of the spectral density and represents the tonal changes in speech over time
8. *Root Mean Square Energy* — Is a measure of the overall signal energy per window and describes the power of the speech signal, a proxy for speaker volume
9. *Zero Crossing Rate* — Measures the number of times the signal amplitude changes from negative to positive and this describes a measure of the shifts in amplitude
10. *Intensity* — Measures the logarithmic change in signal energy over time and tracks the loudness of the speaker

Distilling each of the turn-based features into their different options resulted in 26 distinct features, while extracting speech-based features resulted into 152 additional features, for a total of 178 separate audio features (see Appendix A for the complete list).

### 3.5 Classification Experiments

**Figure 1:**
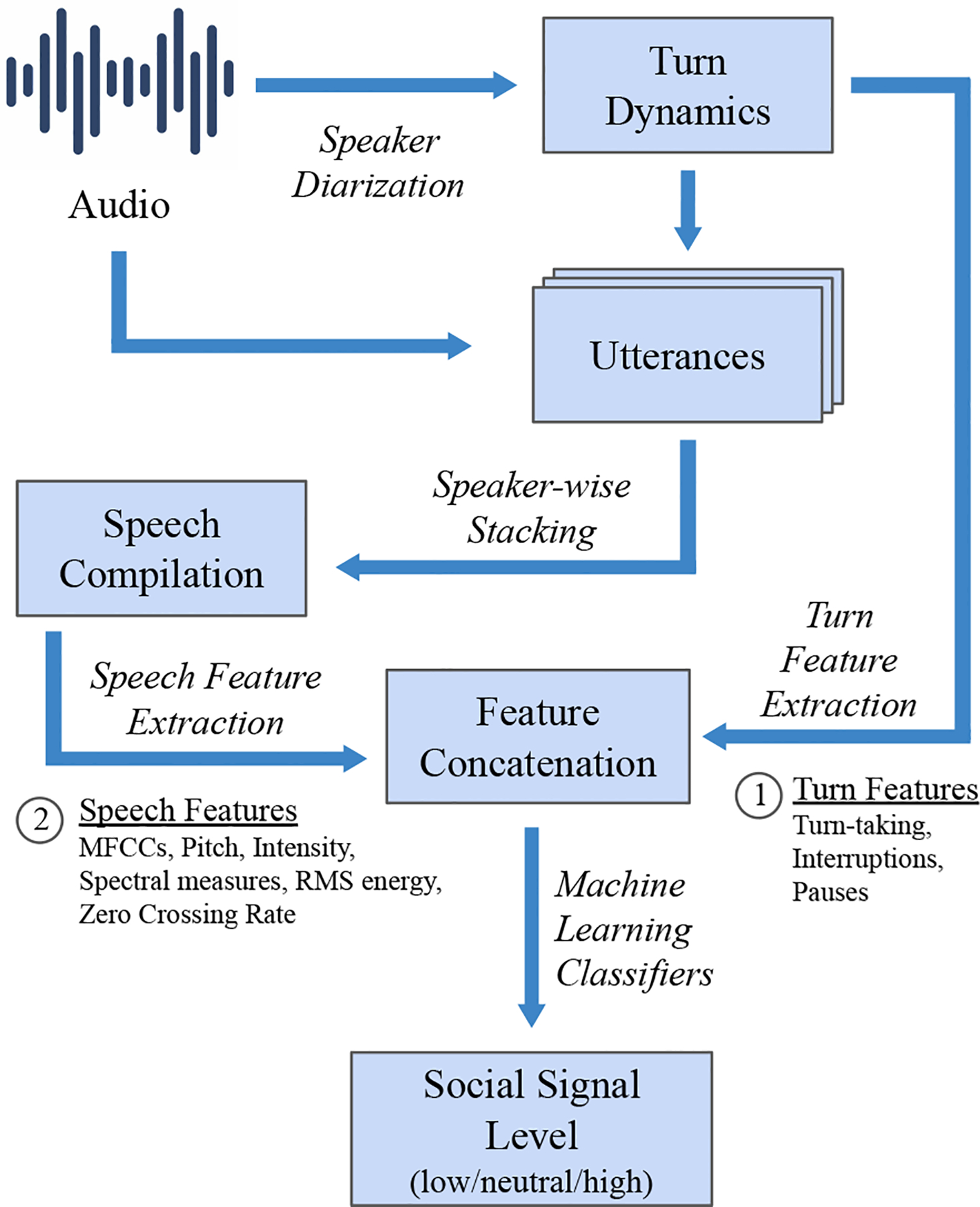
Social Signal Processing Pipeline for extracting turn and speech based features and translating them into social signal levels. This pipeline is used to individually train models for each social signal

Using our feature bank, we use interpretable models to classify the intensity. As evident in Table 1, the *low* case has none or very few samples which cannot be practically useful for modeling. Hence we discard the *low* samples (if any) thereby making the task as *neutral* to *high* separation, which we acknowledge is a limitation. Figure 1 shows the overall summary of our approach. We evaluate the model with a Leave-One-Subject-Out approach and use the average performance across each held out provider as the overall performance. Upon holding out providers each time, we notice that the class imbalance can often increase. For every iteration, we first oversample the training data using the Synthetic Minority Oversampling TEchnique (SMOTE) [23] and then train a series of classifiers.

Among pervasive interpretable models [85] we choose globally interpretable models – Decision Tree Classifier (DTC), Logistic Regression (LR), and Linear Support Vector Machine models & locally interpretable models – SVM with Radial Basis Functions (RBF), Random Forests (RF), and Gradient Boosted Decision Trees (GBDT). Each model is evaluated for a range of hyperparameters optimized using Grid Search. The globally interpretable models are also used to understand if the features are in line with the expectations for social signals in clinics discussed in Section 3.3.

#### Performance Evaluation —

We report on the average across the Leave-One-Subject-Out cross-validation in Table 2. From our validation experiments we use the models highlighted in bold as the final model for the social signal. We optimize for F1 scores for each model due to their robustness to class imbalance [73]. From averaging repeated experiments, we mark that our system takes 6 minutes to process a 15 minute long interaction on an Intel(R) Xeon(R) CPU E3–1225 suggesting its near real-time performance on limited compute power. Since the features are calculated and processed in chunks of audio stream, the system inherently does not require storage of audio files.

## 4 CONVERSENSE TOOL

We build up on the Social Signal Processing pipelines introduced in the previous section and introduce a new interactive web-based system we developed, *ConverSense*, that can be used by providers as an interface to view their communication patterns.

### 4.1 Design Choices

**Figure 2:**
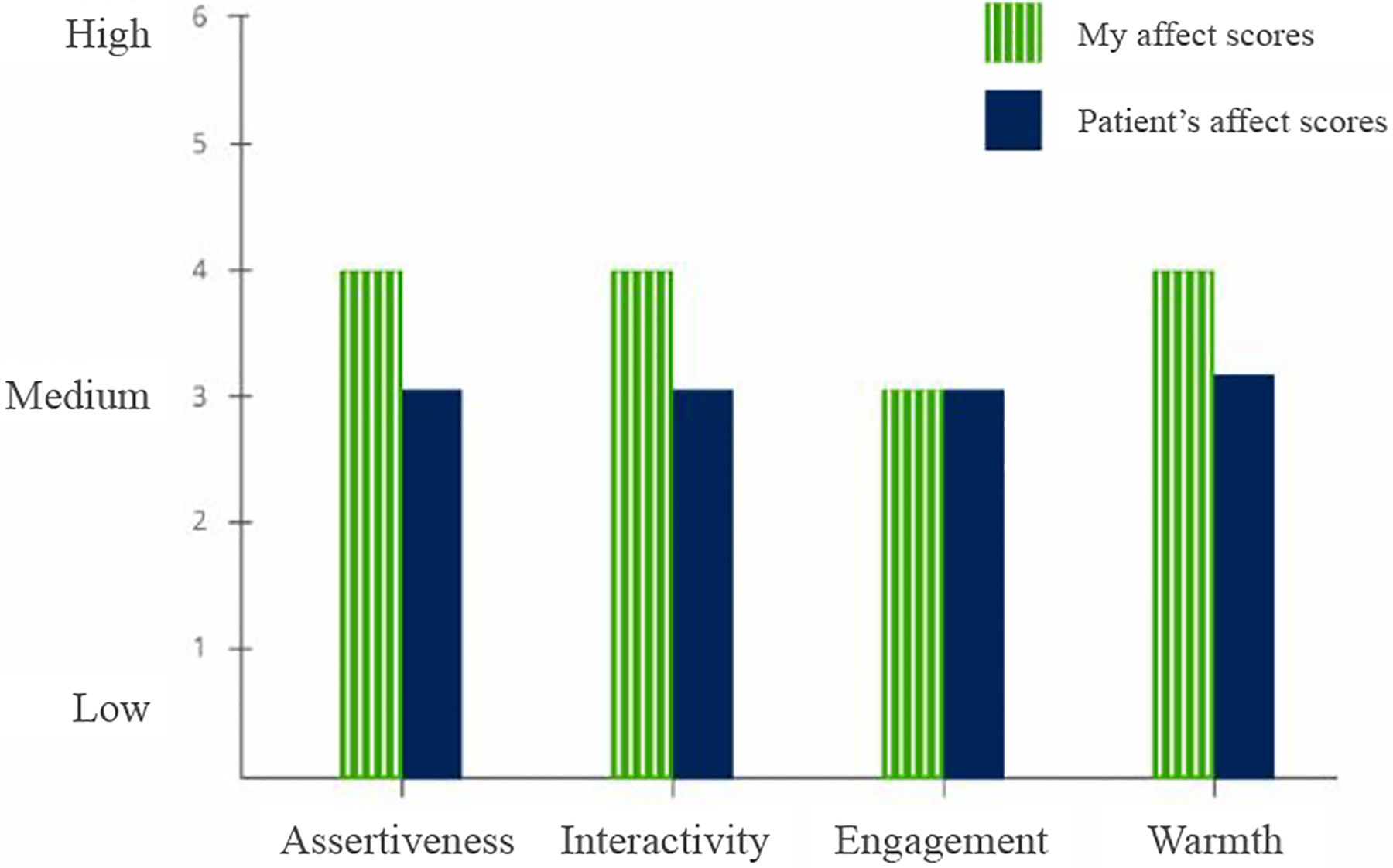
Breakdown of Overall Affect into the four dimensions: Dominance, Interactiveness, Engagement and Warmth

**Figure 3:**
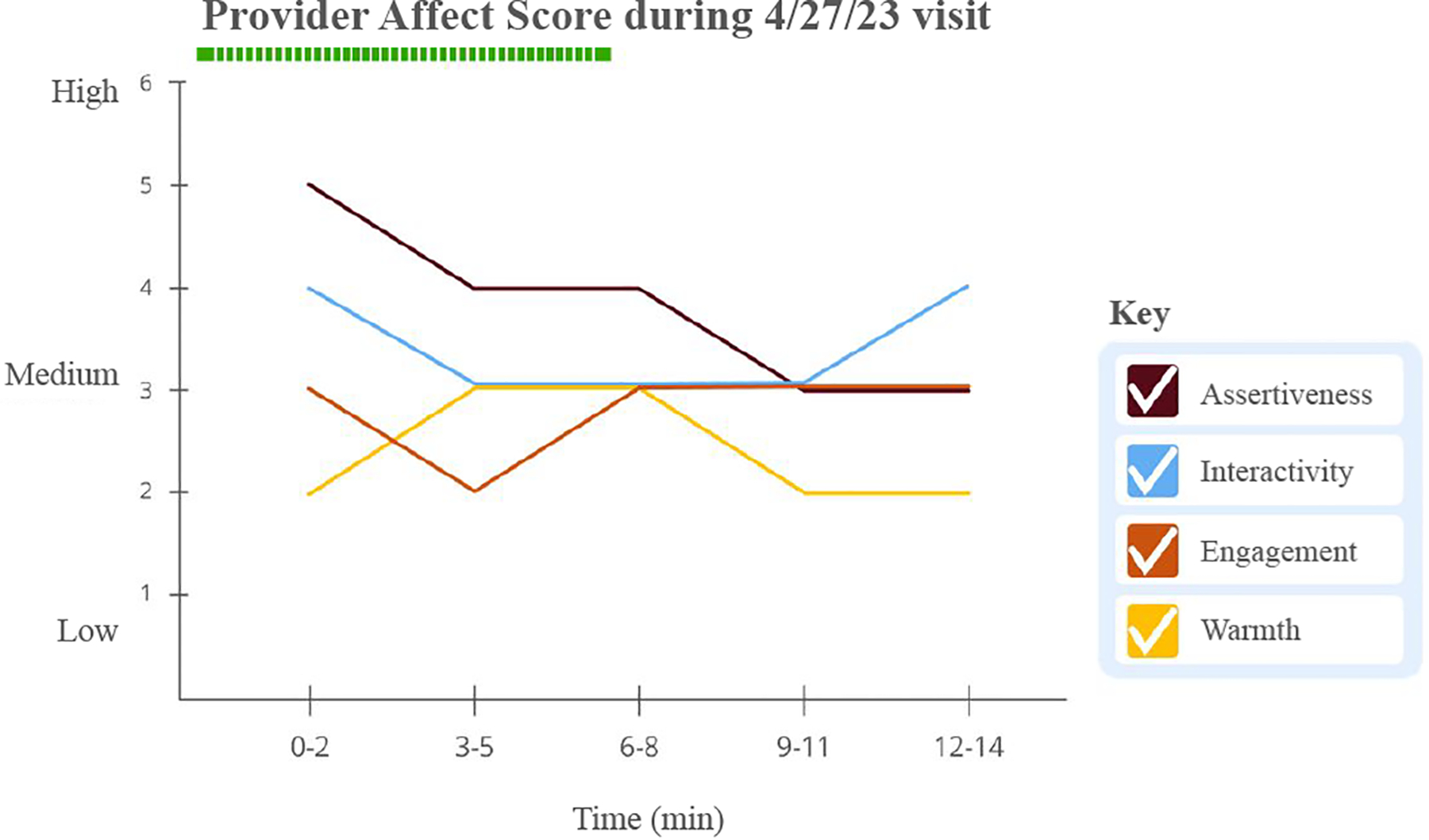
Breakdown of social signals for each visit over time

**Figure 4:**
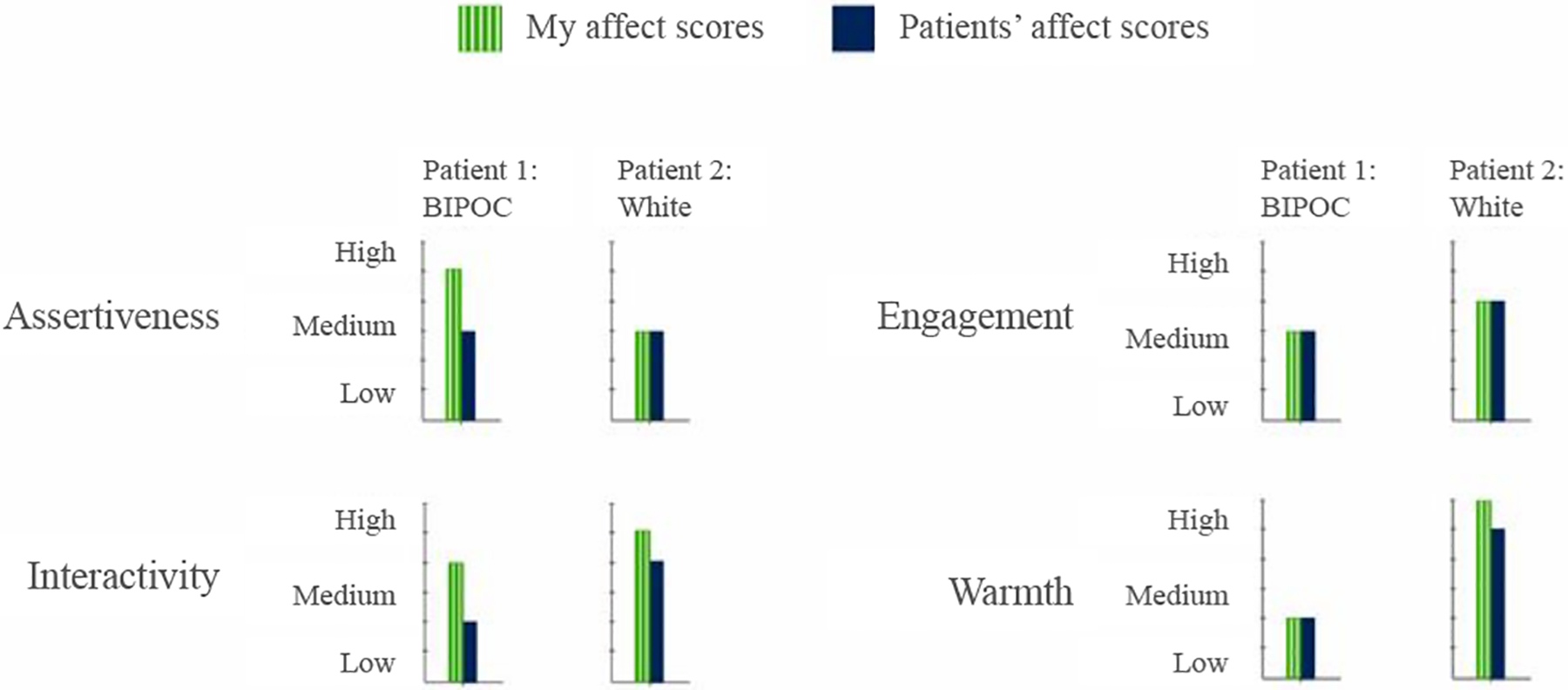
Comparison plots of breakdown between two patients of a given provider

To summarize development of the *ConverSense* tool, we first describe design choices we made to develop a provider-facing dashboard based on our recent formative work on designing communication feedback for providers [4, 31], as well as from direct interaction with the medical expert in our research team.^1^ Next, we describe each design choice with example visualizations (Figs. 2, 3, 4).

#### Overall average affect Scores —

For cues, especially those dependent on both the speakers (e.g. interruptions), insights about a conversation depend upon the circumstances of the visit which get forgotten when analyzing a visit post-hoc [4, 33]. In contrast to illustrating non-verbal cues [1, 33] our work focuses on visualizing communication patterns as social signals [4, 5, 45]. Our approach abstracts *affect* by showing the average of the social signal scores for a person across four dimensions: *dominance, interactiveness, engagement, and warmth*, a subset of the RIAS GAR. We use it as a proxy for overall positive affect in the behavior.

#### Breakdown of Overall Affect Score into dimensions —

To have a comprehensive understanding of their interactions, providers should be able to visualize behavior of their patients alongside their own behavior. This also helps address providers’ need to understand patterns for dip in affect in their communication behavior and if a situation warranted certain response [4]. To uncover patterns, we break-down summaries of social signals across the overall visit for both patient and provider (Figure 2). This approach allow us to show the average rating for each of the four social signals (*dominance, interactiveness, engagement, warmth*) during the visit, thereby enabling comparison at an affect level. We use a bar chart for its simplicity in describing the data in context.

#### Change in affect scores over the course of a visit —

Providers have reported the need to gain context within a visit to better receive feedback [33]. We leverage the slice level scores (in our work, for every three minutes) to visualize change in behavior over time. Such an approach allows providers to introspect on the details of certain segments of their conversation and inspect how each social signal changes across the entire duration of the visit. We use a line chart to show the change in a given time series data for every social signal (Figure 3). The legend should be interactive such that the providers can check/uncheck different signals.

#### Comparing affect scores between patient visits —

Comparing two patients individually might help providers discover if their interactions have similarities or differences between specific individuals, thereby unlocking insights variation in communication style. Figure 4 shows how this can be accomplished by visualizing each social signal and how it changes for two distinct patients across the entire duration of the visit. We show the comparison as a high-level summary across the two patients, and place the resulting bar charts side-by-side to support direct comparison of patient and provider affect in distinct patient visits.

#### Interactions across patient demographics —

Beyond assessments of individual patients, and direct comparison between two patients, we believe that visualizing the data at the aggregate level can allow providers to identify whether they behave differently with patients from different demographic backgrounds, thereby discovering potential implicit biases. For example, viewing interactions across patient visits should allow providers to compare their behavior across different demographic groups (e.g white patients and BIPOC (black, indigenous and people of color) patients). Visualizing behaviors by patient demographics might help to uncover potential implicit biases that have been known to associate with social signals [26].

### 4.2 ConverSense Dashboard Design

In this section we describe the development of a web interface for ConverSense. The web interface was integrated into a web app, developed in Flask,^2^ a python based web development framework. We used Plotly^3^ to support interactive plots, for the various interaction features it supports on the user-end (e.g. zoom, axis-scaling, interactive legend, etc.). While our pipeline does not need to save any audio to process the conversations and generate the social signals, for the lab study, files were saved for future qualitative analysis. The SSP pipeline works seamlessly with streamed audio without the need of saving any audio files.

To help providers reflect on their communication patterns within and across patient visits, the dashboard presents specific visualizations (or “views”), including social signals within individual patient visits, comparison of social signals across patient visits, and population level summaries. We chose a red-blue color scheme that differentiates the provider and the patient. Since Red-Green and Yellow-Blue are highly prevalent color vision deficiencies,^4^ our choice of colors aims for better accessibility. We cluster our labels to allow for automated analysis (Section 3.3), and when re-expanding the signals to the levels that are expected by RIAS (1–6), we map low affect to 1.5, neutral to 3, and high affect to 5 for each social signal.

**Figure 5:**
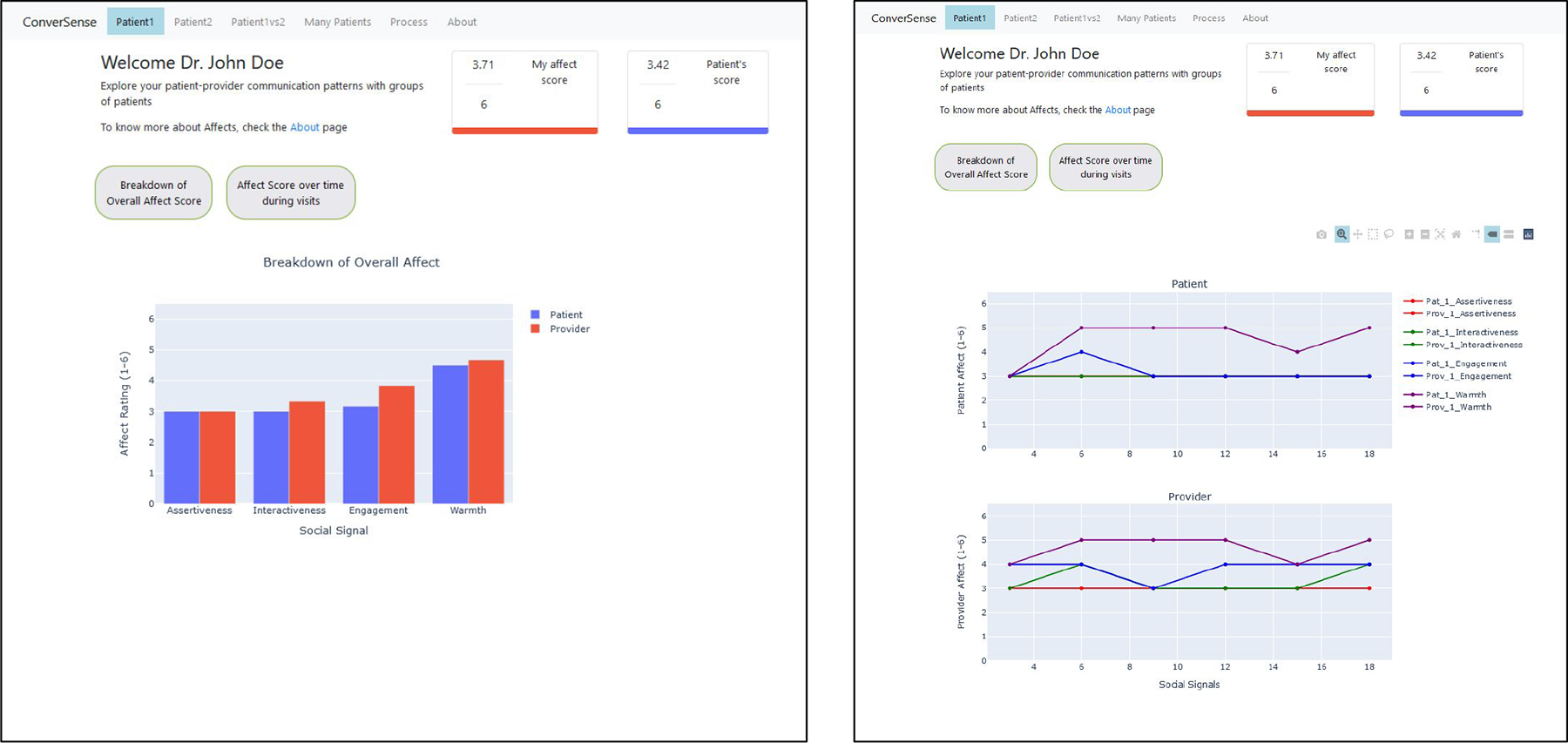
Individual patient visit view. Users can see the overall affect score, its overall breakdown and affect changes over time

**Figure 6:**
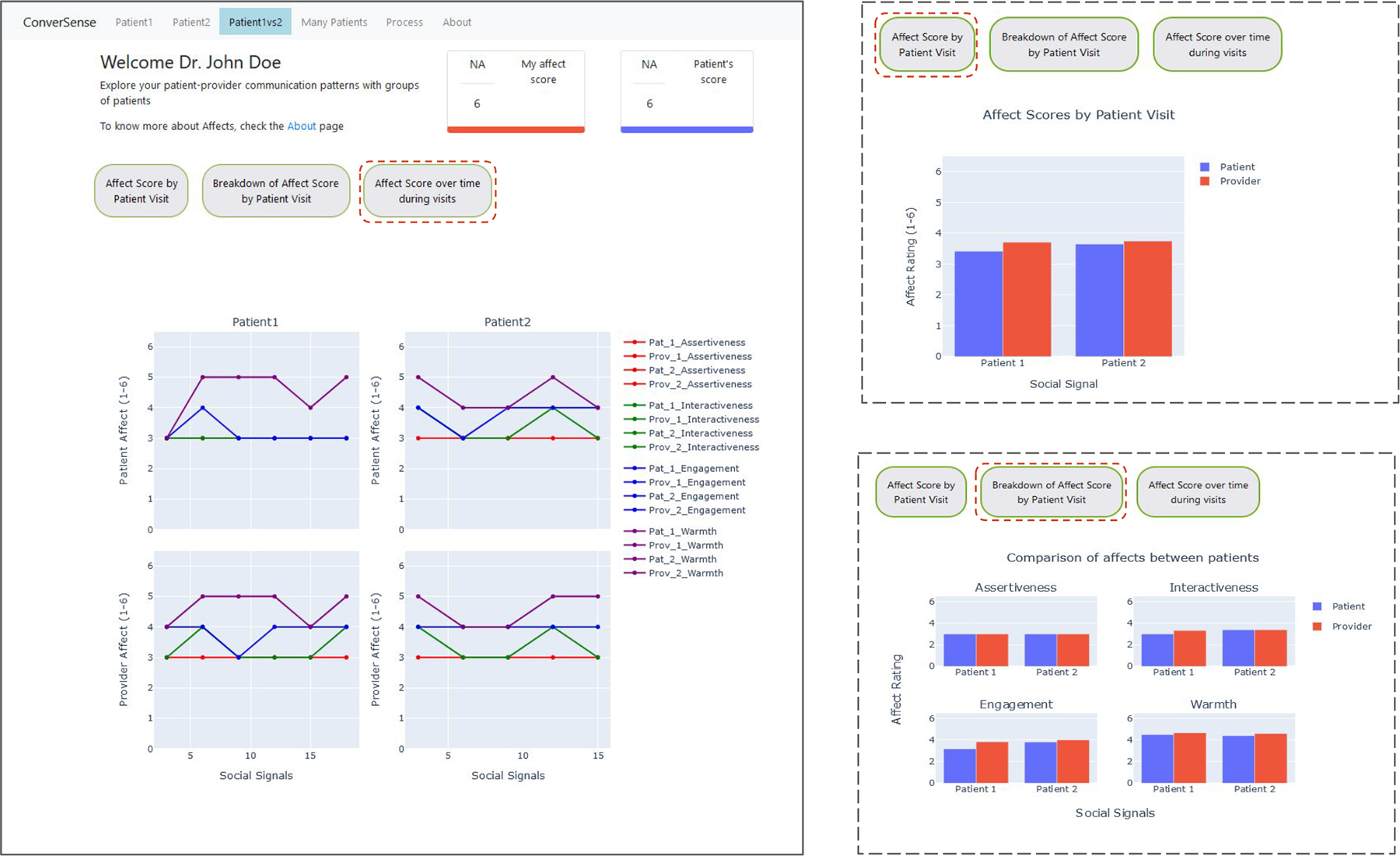
Visit comparison view. Users can see their affective behavior for different patient visits. Image on the left shows the third view. Images on the right show comparisons between patients by overall and individual affects

**Figure 7:**
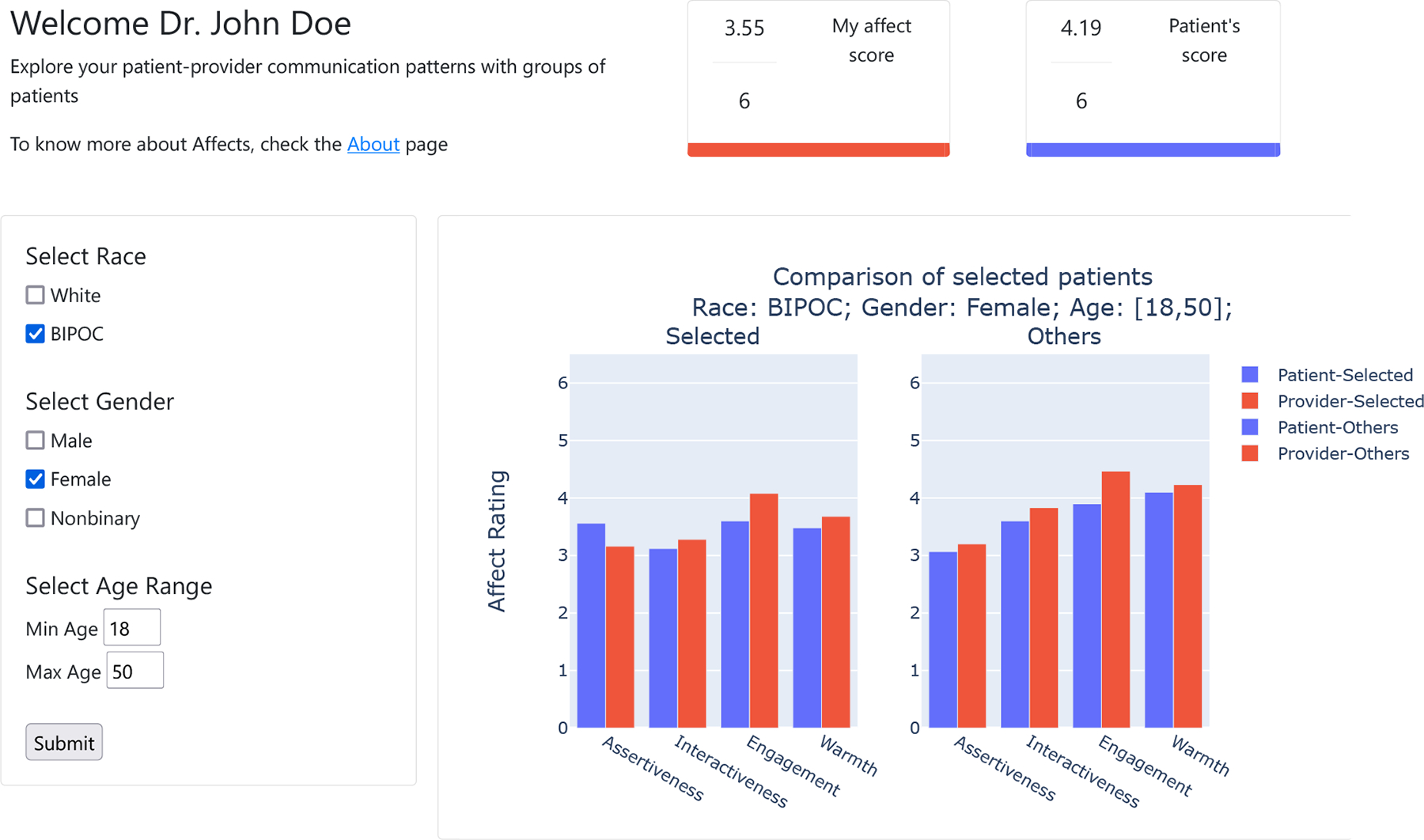
Population view provides different filters to visualize visits with patients from different demographic groups. Users can select filters to visualize the average affect with a selected group compared to other patients

We describe below the specific screens/views developed for ConverSense to support different interactions, and outline them in Figures 5, 6, 7.

#### Individual Patient visit view —

The individual patient visit view (Figure 5) allows providers to view a summary of social signals from their interactions with a given patient. This view is broken down by three components: the average affect score (mean of all social signals over the duration of the visit), affect score broken down by each social signal, and a line chart that visualizes social signals over time.

#### Visit comparison view —

As shown in Figure 6, ConverSense’s Comparison view allows the user (provider) to compare social signals from two different patient visits. This allows them to identify each individual visit and how their interactions varied over time for each. The view is designed to assist providers in understanding individual differences. For example, an angry patient versus a distressed patient or a talkative patient versus an introverted one, etc. To compare the two patient visits, we plot the overall average affect score for the patients and the provider in both visits. We also plot each social signal averaged over time for both the visits. The visit comparison view further visualizes social signals over time in line charts for both patients, side by side. The interactive legend uses a shared axis to support comparative visualizations.

#### Population view —

ConverSense can also help providers identify if their communication show interesting patterns in aggregate across visits, particularly based on patient demographics. Figure 7 shows the population view; users can set filters based on gender, race, and age. Similar to the individual dashboard, we show the average affect score for all patients in the selected filters. We then present a comparative plot between patients of the chosen filters compared with all other patients the user (provider) has interacted with. Since the visualization involves a summary of different visits, which may often vary in terms of progression of behaviors over time, we do not include an averaged time-series visualization in this dashboard.

#### “About” Page —

Describing affect, social signals and non-verbal behaviors can be complex and often contains jargon. Based on prior work and feedback from clinicians [4, 31], it was evident that users need a way to quickly understand the concepts underlying the dashboard. The *About Page* shows a definition for each social signal (described in Table 3), and their relationship with patient-centered care. In addition to the definitions, we describe how RIAS is scored. We designed this page to assist providers in understanding the RIAS framework used by ConverSense.

## 5 EVALUATING CONVERSENSE IN CONTEXT: A LAB STUDY

In this section, we describe the user study conducted with primary care providers to validate our understanding of behaviors in clinical settings, and explore potential opportunities and barriers they perceive in using *ConverSense* in their everyday workflow. In particular, our study focuses on the following guiding questions:

1. How do providers perceive the utility of ConverSense, as a data-driven communication feedback tool?
2. How do providers perceive social signals as an approach for communication feedback?

### 5.1 Study Design and Participants

The *ConverSense* system consists of two primary components, the SSP backend and the visualization tool. The backend analyzes conversations and generates affect scores for four social signals (dominance, interactiveness, engagement, warmth).

We investigate providers’ perceptions of the utility of ConverSense and provider’s approach to social signals as a communication feedback mechanism, we studied how ConverSense could possibly integrate into their day to day practices. We recruited a convenience sample of five practicing primary care providers, P1-P5 (see Table 4), through word of mouth and the authors’ professional network within our healthcare system, and asked them to participate in simulated patient visits where they interacted with patient actors following scripted scenarios. All participants were physicians (i.e., MD) with healthcare experience ranging from 4 to 32 years.

After seeing the patients, we used ConverSense to show participants communication patterns from the simulated visits. To enable participants to compare communication patterns between individual patient visits, we conducted two visits per participating provider, each with a different patient actor. In addition to social signals generated by ConverSense from each visit, we enriched the ConverSense with additional mock patient-visit data, so that providers could explore the use of ConverSense’s population view.

We designed a semi-naturalistic environment for providers by creating a mock study room resembling a clinic visit room, with the addition of ConverSense recording equipment. Further we standardized simulated visits across participants to understand the impact of ConverSense. We took into account three major patient factors that can bring about variable provider behaviors: (1) Difference in appearance or demographics of the patient [42, 82], individual patient behavior [40] and the reason for the visit [81]. Adapted from scenarios originally designed as “racially triggering” and with permission from the lead author Kanter et al. [52], we scripted two case scenarios with subtle cues related to racial bias for two common health issues that show historical healthcare inequities: stereotypes about compliance with diabetes care [70] and treatment for chronic pain [47]. The patient actors, one black and one white, followed these scripts during simulated visits so that they acted in a prescribed manner regardless of the flow of the conversation. To control for the reason of their visit, each scenario covered a specific case, symptoms, and history with a script that patient actors were asked to rehearse beforehand (see Appendix B). To counter for differences in appearance, we ensured that the majority of the physical traits of the patients were similar except for race.

### 5.2 Study Protocol

The study was conducted as a 90-minute session and study procedures were approved by our Institutional Review Board (IRB). Providers were welcomed in a “back-end” room equipped with a computer, where all of their non-patient facing interactions with the study team occurred. The “study room” was reserved for simulated visits, and the provider was directed to it where the patient actor was sitting and ready for their visit. Sessions were comprised of four parts:

1. **Pre-Study Interview:** The provider first underwent a brief structured interview to describe their demographics, healthcare qualifications and experience, and methods used in their clinic (if any) to monitor communication patterns. During the pre-study interview, the first patient-actor set up in the study room, to prevent any interaction with the provider before the simulated visits started.
2. **Simulated visits:** For each of the two case scenarios, the provider was then told that they had approximately 15 minutes to attend to the patient. Each visit was audio recorded for analysis by our ConverSense pipeline and video-recorded only for further analysis by our research team. After the first visit, the provider was asked to take a 10 minute break in the back-end room, while the second patient actor was set-up in the study room to repeat this process for the second simulated visit. The provider then interacted with the second patient actor for 15 minutes followed by a 10 minute break. This break also provided time for the research staff to upload recorded interactions to process through the ConverSense pipeline.
3. **Post-visit feedback and interview:** After completing the two visits a member of the research team presented the participant with the ConverSense tool and providers were asked to review their communication patterns in a semi-structured manner for roughly 30 minutes. The research team explained that the scores were AI generated and trained based on expert opinions to understand user perceptions of the backend. Providers interacted with the ConverSense web tool and explored different functions on their own (with researcher guidance available) and were then asked to “think-aloud” [32] to describe their actions. Next, providers responded to semi-structured interview questions about their experience with the visits, impressions about the utility of the ConverSense tool, and perceptions about challenges and opportunities for using social signals as an approach for communication feedback for healthcare visits..
4. **Post-visit survey:** Finally, the provider completed a post-study survey about the utility of ConverSense that included the NASA Task Load Index (TLX) focused on 6 dimensions of cognitive load (i.e., mental demand, physical demand, temporal demand, effort, performance, and frustration level) [44], and the System Usability Scale (SUS) [11] on perceived usability, including learnability. Upon completion of the post-visit survey, clinicians were offered optional additional time for any other questions or feedback.

### 5.3 Data Analysis

From our study we collected various types of data such as participant characteristics from the pre-interviews, the video recorded simulated visits, screen recording of participants’ interaction with ConverSense during the post-visit feedback sessions, recording of the semi-structured post-visit interviews, and ratings from NASA TLX and SUS surveys. Below we discuss the analysis steps. We analyzed the data using a mixed methods approach.

1. **Pre-study Interview:** Participant preliminary data was converted to counts and summarized with descriptive statistics. Qualitative information was tagged using an inductive approach.
2. **Simulated Patient Visits:** Each visit was coded using RIAS (Section 3.2) protocols. ConverSense predictions were statistically compared with coded scores.
3. **Tool Interaction:** Screen recordings of the providers’ interaction with ConverSense were quantitatively scored for use using BORIS [36] and each participant interaction was coded in terms of active engagement with graphs, and views based on number of page flips and time spent viewing a page.
4. **Post-Visit Feedback:** Qualitative analysis was done using an inductive approach by iteratively coding participant responses to interview questions in reference to the utility of ConverSense and the use of social signals for communication feedback. The post-visit surveys were scored according to published guidelines [11, 44] and summarized with descriptive statistics.

## 6 RESULTS

This section discusses the results of the study through usability surveys, quantitative analysis and qualitative analysis. We describe our findings to validate our considerations and design decisions. We will use the convention ($\bar{x}=\_\_$, *σ* = __*)* for reporting the mean and the standard deviation for any quantity.

### 6.1 Utility of ConverSense as a Data-driven Communication Feedback Tool

#### Data-driven communication feedback helps providers evaluate patient interactions objectively —

All five participants liked the idea of using a data-driven visualization to uncover their communication patterns with patients. Participants reported how current methods employed in clinical practices (if any), such *patient-reports* [P2, P3], *physician coaches*[P2], don’t provide contextualized feedback for each visit and hence are not comprehensive enough to fully understand patterns. P3 also expressed how he sometimes evaluates trainees’ patient interactions and provides them with feedback, and that having more data-driven feedback would help this process. P5 explained that his peers use methods such as self-reflection without data, which can be subjective and prone to *their own biases*. Patient reports of provider communication behaviors are also typically *anonymized and shared in summaries monthly or quarterly*, which becomes hard to understand really *“who was the patient or which interaction it was”* [P2], making the actionability of these surveys poor.

#### The proposed approach has potential to generalize to the real-world —

Our model was trained on samples from the EF dataset [62]. To validate the model in an “out of sample” setting, we test it on unseen data. We manually coded each interaction from the Lab Study 5 (n=10), using a protocol similar to Section 3.2. From our post-hoc analysis we see that our pipeline had a mean normalized accuracy per visit of 0.94, 0.77, 0.89, 0.95 for dominance, interactiveness, engagement, warmth. This shows that the models are capable to perform reasonably even on unseen data from a different distribution.

#### Computational approaches could help reduce observer bias —

Compared to their existing methods of communication feedback, which are largely subjective in nature, participants found merits in the ConverSense computational approach for self-reflecting on one’s own implicit bias. Human observers, such as providers or physician coaches, can offer *impactful personalized feedback* due to their expertise and experience, but they will always carry some bias shared by their experiences. One of our participants remarked that the ConverSense computational approach *“is independent of overt or underlying bias and can [thus] provide more accurate insights in complex, individualized patient interactions”* [P3]

#### Use in near-real-time and cross-device is valued —

Participants concurred that they would prefer a feedback tool that visualizes their communications post-hoc for review after visits. They argued that real-time notifications during patient visits can be *intimidating* and *distracting*. Three out of five participants [P1, P4, P5] also said that they would ideally review each visit while their memory of the visit is still fresh, potentially at the end of each work day. In contrast, P3, advocated for real-time feedback during visits and sporadic interactions with a communication feedback dashboard. All participants agreed that future tools should be accessible on their computer screens in clinics as well as mobile phones when they are outside of the clinic. These findings may imply that future tools should also work in near real-time, to facilitate this intended use. P2 and P3 also suggested that communication feedback is critical for providers in training, opening up the opportunity to use ConverSense during medical education, with a user group that might amplify the impact of our tool.

#### Interacting with social signals in a data-driven tool is hard, but can be learned —

On average, participants rated the SUS usability of ConverSense to be on average just *OK*, or *marginally acceptable* ($\bar{x}=60.55$*, σ* = 22.17 out of 100). Participants also reported that they would need to learn a lot before using the system ($\bar{x}=3$*, σ* = 1.58 out of 5), but also that they would imagine being able to learn quickly ($\bar{x}=3.6$*, σ* = 1.5). We also learned about their perceptions of cognitive load while using ConverSense from NASA-TLX: participants overall reported low cognitive load when using the system; all participants except P3 reported their overall cognitive load to be below 30 out of 100, with all dimensions except performance to be below 25 out of 100. Performance (level of success in completing tasks) was however rated higher ($\bar{x}=51$*, σ* = 31.50 out of 100), meaning that the load of completing tasks was consistent with the widely-used threshold of 50 [74].

#### Line Charts can become confusing during overlaps —

ConverSense depicts social signal affect scores over time using line charts. Participants however found it confusing when lines overlapped on one-another, as exemplified by P1: *“Why did my warmth signal go missing in this time period?”*. The use of the interactive legend and configurable plot were not very intuitive to the users. For example, during the think aloud exploration, P1 did not discover that the legend was interactive, whereas P2, P4, P5 spent a lot of time selecting and unselecting signals to get a desired configuration and became frustrated with the number of clicks required.

### 6.2 Social Signals as an Approach to Communication Feedback

#### Social Signals alone may not show the complete picture —

All participants agreed that social signals can represent more “nuanced” [P1, P3] communication patterns than non-verbal behavioral cues alone. However these same nuances can make the patterns difficult to fully understand as they were found incomplete descriptors of communication feedback. For example, one participant stated that each person has their own *“definition of [these] social signals”* [P3] and that makes them *“open to various interpretations”* [P1]. Not having knowledge of the system’s interpretation led participants to question the validity of the tool. Their repeated attempts to decipher the definition of each social signal was also visible in the number of flips participants performed from one page to another. Participants clicked to the “About” page approximately once per each view ($\bar{x}=0.99$*, σ* = 0.39) when they were confused. Further, most did not recall the definition later, leading to revisiting the About page multiple times.

#### Providers need more contextualized and actionable feedback —

All the participants mentioned the tool’s inability to provide actionable strategies to improve communication behavior when particular patterns emerge. In particular, P2 was concerned about the inability to act on ConverSense’s feedback and he suggested that the tool could benefit from showing *strengths* and *weakness* in terms of social signals: *“I guess that my warmth must have been low in a certain slice, but the tool never told me how to be warmer”* [P2]. P3 also discussed how the current feedback mechanism lacks *actionable insights*, which a human observer or an evaluator can provide.

Participants also experienced a mismatch of expectations when considering their existing methods of communication feedback by an evaluator (e.g., coach), which can provides more actionable feedback. Reflecting on his own experience as an evaluator, P3 questioned if and how improving on scores for a particular social signal might actually improve patients’ experience;

“Feedback through visualizing social signals deviates from conventional paradigms of assessment feedback [such as visualizing non-verbal gestures]. It does not use discrete behaviors as perceived by the patients that we [come to know] from a patient’s perspective. We don’t have a third eye that is independently gathering information and describing it in domains which are not very discrete, and instead quite ambiguous and uses unfamiliar terms. What exactly is interactiveness? And how do I look at that in a way that might provide commentary on what I do well, or don’t?”

[P3]

Finally, when thinking about how to better understand the specific social signals feedback, participants expressed how they would *“lose memory of a specific interaction over time and having context about that interaction, could help remember the interaction better”* [P5]. Participants also suggested using low affect scores as a mechanism for identifying *“clips”* from the interaction where communication could be improved..

#### Comparing provider and patient affect scores helps in understanding actions and reactions —

Most participants found it useful to compare their affect scores with the scores of patients to gain a relative understanding of the dynamics of the interaction during a visit. They compared the patient scores to their own scores and even tried to correlate it with their memory of the interactions. P5 found the juxtaposition helpful, as it helped them map their own behavior against a global baseline. They said *“I felt pretty warm to her and I thought she was actually much more cold”* [P5] when they saw the patient warmth as similar to theirs. P2 also shared a similar experience and added that *“if an interaction did not go well, I would wonder if it was mine or the patient’s fault”* and seeing patient scores helps them understand why they acted or reacted in a certain manner. However participants also voiced that this approach might result in being more defensive in the interpretation of communication breakdowns, as evidenced by P2: *“they weren’t very warm so I wasn’t too warm to them”*.

#### Absolute affect scores are difficult to interpret —

Participants described the absolute affect scores or the numerical measures of affect throughout the dashboard as difficult to interpret. While the score of “3” for neutral behavior on which RIAS is based, participants were not able to determine what makes for a “good score”. Participants expected a more local benchmark either compared to themselves or with their peers. P1 shared: *“my overall affect is 2.86, is that good or bad? … Having a point of reference, either through self-comparison over time or by comparing to others, would provide clearer context for understanding a score like 2.8”*.

P4 also interpreted the scores as percentages to judge how far he was from “100%”; he found that the choice of using the RIAS denominator (six) was problematic for calculating percentages. Three participants (P1, P2, P4) demonstrated that they fundamentally aspired to achieve the optimal scores, but also acknowledged the perils of chasing numbers in the context of their interactions. P1 described this belief:

“Attaining a score of 6/6 might actually make the providers [to have] excessive empathy, potentially compromising professionalism. A balanced approach, combining rationality and empathy, is crucial for maintaining professional behavior and earning patient trust.”

#### A lack of knowledge of the underlying models might reduce the trust in the system —

There were instances where participants did not agree with the tool’s evaluation and expected their affect scores to be higher than those of the patient. This reduced their trust in the affect scores [P1, P3, P4], with P1 even saying *“2.8 was too low given the number of concessions [she] made”*. Perceptions of mistrust were so strong that participants rated their overall trust in the model only moderate ($\bar{x}=5.20$*, σ* = 1.49) on a scale of 1 to 10. Each participant questioned the interpretability of the SSP models and explained that while the models were based on non-verbal behaviors, they would only be able to trust them if the *knew the specifics*. For example, one participant shared:

“I’m curious about the methods and like, how it came up with these things. Do I think it did a good job? Yeah, I mean, I don’t know was I less warm than the patient was? “

[P4]

Mistrust expressed by participants reflected three dimensions: (1) Validity (do the signals measure true social behavior?) [P1, P3], (2) Performance (how well do the audio features map to the perceived definition of the signal?) [P1, P2, P5], and (3) Interpretability (why did I score so low?) [P2, P4].

## 7 DISCUSSION

The development and deployment of ConverSense yielded a several key insights about the use of AI-generated social signals to characterize and provide feedback to providers on their communication with patients. We built a SSP pipeline that ingests audio-streams of conversations and tracks the magnitude of four social-signals: dominance, interactivity, engagement, and warmth. We embedded this pipeline into ConverSense, a web-application for providers to visualize their communication patterns, both within and across visits. Our user study with clinicians demonstrates the value of ConverSense to provide feedback on communication challenges, and the need for this feedback to be contextualized within the visit. Through this novel approach that uses data-driven self-reflection, ConverSense can help providers improve their communication with patients. We discuss below, the implications of introducing ConverSense and the opportunities they present.

### 7.1 Beyond the Status Quo

Through the creation of the ConverSense system, we bridge several gaps in the prior work that we have introduced in Section 2.

First, compared to traditional manual analysis of patient-provider interaction, ConverSense introduces the first Social Signal Processing (SSP) pipeline that uses machine learning to predict the RIAS signals of dominance, interactivity, engagement and warmth. Our web tool integrates the machine learning pipeline to present social signals and allows different comparisons, an aspect largely lacking in existing systems that instead focused on presenting raw communication behaviors [33, 58]. By designing the tool to provide post-visit near real-time feedback to the providers instead of real-time [33, 45], our novel approach for communication improvement facilitates self-reflection among providers. Our lab study found that participating providers were interested in the idea of long-term communication feedback. They also appreciated a monitoring system that uncovered various aspects of their social signals. Overall ConverSense sparked new ideas in providers for better self-reflection to improve upon their communication.

Second, we discussed in Section 2 how existing SSP systems focus on non-verbal cues [33, 58] that fail to capture the important socioemotional context of patient-provider interactions. Due to being rich indicators of clinical social context [45, 78], we adjudged social signals to be the missing context. However, prior work that characterizes social signals in patient-provider interaction has relied on manual coding by a trained observer, which is difficult to scale. We addressed these challenges by introducing a novel end-to-end SSP pipeline that tracks social signals in near real-time and can generate feedback for post-visit review through a visual dashboard

### 7.2 Enabling new forms of computational interactions with social signals in clinics

While the impact of employing RIAS to characterize medical interactions has been massive [26, 41] with ConverSense, computationally sensing four key RIAS dimensions is now possible. Our introduction of the SSP pipeline showed improved performance when validated against the new visits recorded in the lab study. Despite our intentional choice for ConverSense to be unimodal (audio-only), this finding demonstrates how the system’s capability compares to similar tools that require multiple modalities, but introduce a number of logistical hurdles. In addition, our specific modeling approaches (described in Section 3.4) to transform raw signals to RIAS signals through non-verbal cues, can further open the way to a variety of novel machine learning developments, geared to sense more signals, with better granularity, and new input modalities, such as video and transcripts. Our work also adds to available practices for providing feedback to providers on their medical interactions, such as direct feedback from communication coaches or attending physicians (senior providers) by offering the ability to track every patient visit through self-reflection. This enhances what can be done through human coaching introducing a scalable model that can’t be achieved by human coaches. An important focus of future tools should be on empowering providers to engage themselves with their data and providing actionable and personalized “quick tips” for improvement [31].

### 7.3 Social signals can motivate the design of future feedback systems

In contrast to prior work [33, 45, 58], we used RIAS-based social signals as a mechanism for visualization using simple charts that made the system easy-to-learn. Through our approach we found that absolute values of affect helped providers find a measure of their behavior such that they sought to find the most optimal score and work towards achieving it. Upon further observation we attributed these ideas to the fact that our tool design (Section 4) enables comparisons at various levels. These comparisons can be made by the provider with data collected across different patients’ visits, but providers also sought comparisons beyond their own patients, comparing themselves with their peers. This suggests opportunities to introduce friendly *competitions* to achieve the best communicative behavior, a training mechanism that could be explored in future work.

While competitions can promote behavior change through peers, there are equivalent benefits to self-improvement [24]. Competition and individual goal-setting can essentially be described as two means towards completing a task [19]. In our lab study, some providers perceived social signals as their current state and aimed to work towards achieving a certain goal. For example, P2 asked how he could become a warmer version of himself. We propose that social signals can act as a driving mechanism for *goal-setting*. Most goal-setting frameworks work towards improving a derived measure of performance that can change over time. Given their relation to overall affect, social signals can be effectively used as the measure to be optimized for. For example, the transtheoretical model (TTM) [75] for goal setting envisions behavior change through six distinct stages – precontemplation, contemplation, preparation, action, maintenance and termination. In a clinical setting, the use of tools like ConverSense places providers already in a state of preparation to act on specific feedback. Utilizing a social signal based data-driven feedback like the one we introduced, could propel users towards action stage such that the goals can be driven by the overall affect scores.

### 7.4 Need for better understanding social signals across different user groups

An overall theme in our findings is the difficulty to really understand how RIAS social signals directly link to patient-centered clinical practice and specific interpersonal interactions with patients. To that extent we offer three suggestions for standardizing social signals.

#### Towards alignment of definitions of social signals —

From our semi-structured interviews with providers, we learned that despite our SSP models are an abstraction of traditional RIAS social signals, and are based on patient-centered communication concepts such as empathy, these signals can be susceptible to differences in interpretation.

While this is a valid concern, we believe it is very much due to a missing common ground. In fact, our training dataset (Section 3.2) was based on coders’ subjective judgements of RIAS dimensions after achieving inter-coder reliability, and the feature extraction process for the SSP pipeline was based on known interpretable vocal non-verbal behaviors that map to social signals associated with patient-centered communication. This is clearly not enough, and we believe that any tools that look at assessing patient provider communication should ensure *alignment of concepts* between the tool and the user. Our objective and interpretable approach already forms the basis for future work in this direction: in case of non-verbal behavioral pipelines, one way to align SSP definitions with user perceptions is to integrate exemplar interactions when training users in use of the tool. Videos sampled from datasets with widely agreed definitions of social signal levels such as [78, 82] could be added as examples to ConverSense and wrapped in a new *“Learning Resources”* module. Such a module could also help address the need participants expressed for actionable examples on how to improve communication behaviors (i.e., “What makes for a “good score”). Before interacting with the dashboards, users could explore these foundational resources align their interpretation with definitions used.

#### Benchmarks for baseline comparison —

Following RIAS, we set a score of “3” (global average or neutral starting point in RIAS) for neutral behavior during the coding process. However, the meaning of what a baseline is may vary across users based on clinical and/or socio-cultural experiences of providers, as was evident in our user study in which participants expressed confusion regarding baselines and comparison of scores on the 1–6 scale. Building on participants’ suggestions, we propose to add another time series with each plot, describing the local average score for each social signal or overall affect. Introducing a local baseline in addition to the neutral point of “3”, can help provide meaningful benchmarks. With adequate visit data, local benchmarks could be derived over a number of factors, such as the individual providers, a particular clinic or geographic region, or even specific patient cohorts.

In addition to providing comparative benchmarks, future work could build on recent work around gamification [18, 69, 90] that suggest that viewing ranking with respect to peers can improve learning outcomes. Additional metrics describing a provider’s rank with respect to their peers could be introduced as a benchmark. While this might create a positive/healthy competition, it can also have negative consequences; a deeper dive into such an approach is required to weigh the pros against the cons.

#### Complementing social signals with non-verbal communication feedback —

We learned that actionable feedback is critical for providers, but that receiving social signals, while valued, is not sufficient. In contrast with recent work [4], providers also mentioned that they would have liked more information about the visit be presented in ConverSense about their non-verbal behaviors (i.e., number of interruptions, pauses, total talk time, etc.). Our findings indicate that the combination of (1) social signals to identify specific breakdowns during the interaction, and (2) information about non-verbal behaviors during that specific time slice, could provide a more complete picture that when combined with education resources, could facilitate more actionable feedback.

Give that our SSP models are interpretable, we fortunately can easily trace back to the underlying nonverbal features and incorporate this information into a rule-based feedback system. Since our features hold associations towards non-verbal vocal cues, we can estimate how close the speakers are towards boundaries/thresholds and provide specific and actionable insights for making their communication more centered. For example, Figure 9 shows an example of a Decision Tree that uses vocal cues to rank warmth as neutral or high. Someone who aspires to increase their warmth, would have to either allow patients a higher talk time (help patients to feel heard), or be more responsive when there is a change in speaking turns. Potential feedback could include recommended behaviors to drive these improvements, such as the use of back channelling (e.g., “uh huh”) to show interest or allowing the patient to have the floor to speak.

### 7.5 Visualizing and interacting with pivotal moments of conversations through non-verbal cues

We found that social signals provide value as descriptors of change in affect, but that the providers we engaged with expected a more actionable form of feedback. Based on their suggestions, we propose to enrich ConverSense to identify regions of low affect scores as potential *pivotal moments* of an interaction. P2 proposed embedding “clips” of the recorded interaction to allow users to better understand the context of these interactions. Such an approach has been described as both auto-confrontation [64] and stimulated recall [59], and studies show that bringing people back to previous interaction by showing them what happened at a particular time can stimulate episodic recall. While clips of pivotal moments might help jog the memory of users about past interactions [59, 64], we learned that providing additional data about non-verbal behaviors alongside the social signals can also help.

**Figure 8:**
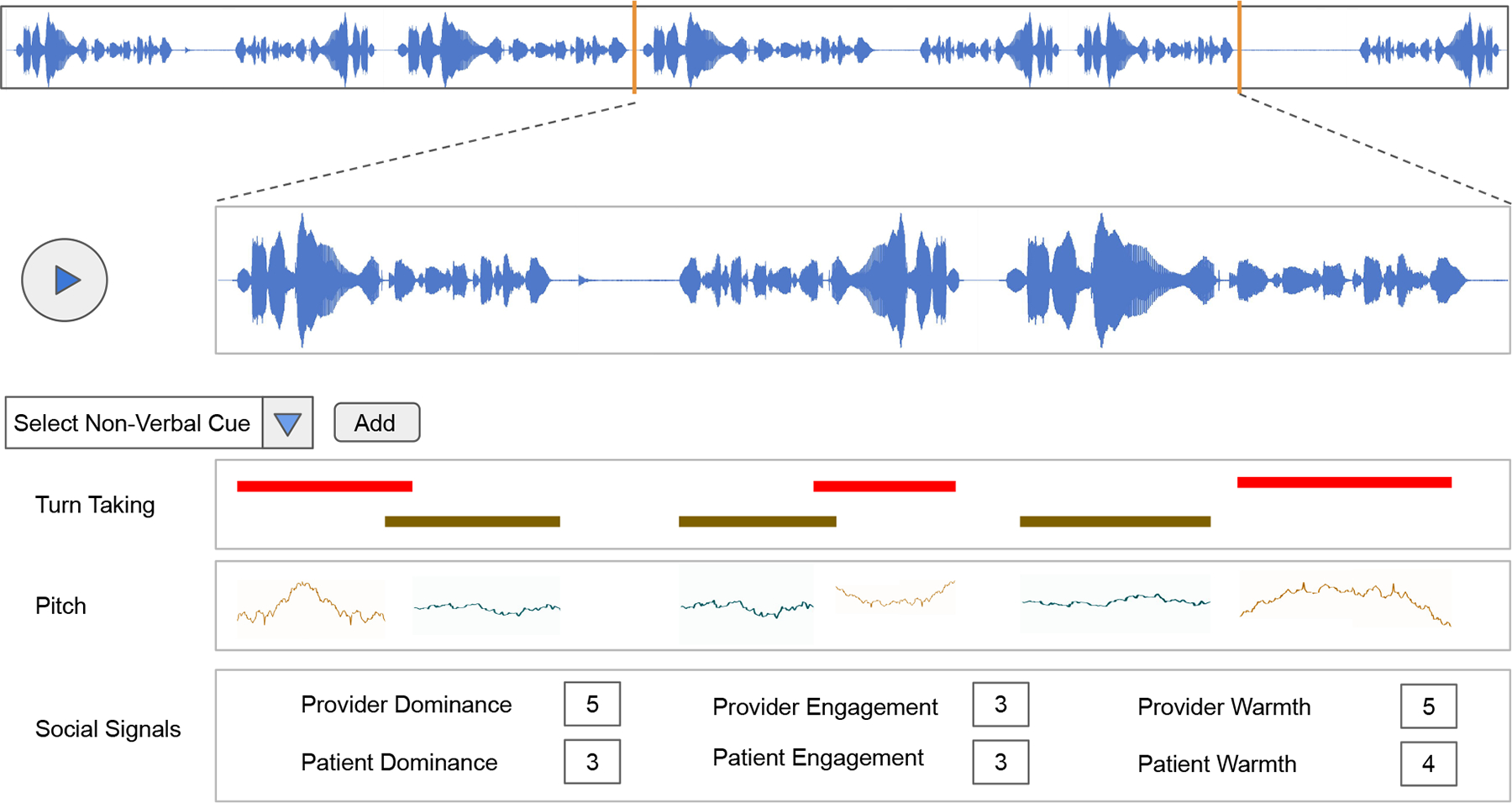
Proposed Visualization Dashboard for additional context about the interaction

Chronoviz [35] has popularized visualizing multimodal data of user interaction alongside video recording of them, and has been used to show video clips, turn-taking, hand gestures and other behaviors in patient-provider interactions [101]. We show in Fig. 8 a proposed visualization scheme that adapts this concept to ConverSense and introduces new contextualized feedback mechanisms as described by study participants: users can step through the clinical recording (in our case, pure audio waveforms) and select filters for different cues they want to visualize over time. The use of graphical imagery has been a successful mechanism in communication feedback systems [2, 33, 45, 83]. As we have seen in the qualitative interviews of our lab sudy, in the context of post-hoc visualization these cues can often get overwhelming for an entire visit and the pivotal moments can potentially help reduce the cognitive load in such cases.

Similar to recent multimodal interaction approaches, we can model the non-verbal cues as an anomaly detection task [1] to identify regions of importance in the visit. While RIAS would require the full three minutes, these regions can provide more fine-grained highlights to help providers review interactions faster.

Our participants wanted more context within a visit and needed a pervasive structure to interpret social signals in each slice. We believe that visualizing their cues can also help them align better with their social signals as outlined by ConverSense. Further, being able to replay selected clips might further aid in more contextualized self reflection.

### 7.6 Towards a human-centered AI approach by improving user trust

One expected tension that we tried to address with this work is the relationship between provider trust and AI-based decision support tools. It is well-known that lack of knowledge about underlying machine learning models and their inner parameters diminishes the trust in any autonomous system [30, 76]. While we have tried to address this in ConverSense by using fully interpretable models (i.e., such that change in each signal can be traced back the the cues related to it), providers were not able to fully engage with our models, as outlined by [P1]: *“Well, not knowing what how these are calculated [is a big concern]… [feels] a bit strange. Why does it say I was not warm to the patient? They were really out of their mind.”*. The lack of trust, or better the inability to engage with interpretability, became an additional obstacle for our providers. Recent work on imprvoing trust in models through visualization [22] prescribes different aspects of visualization that can impact trust. Future systems can refer to such design guidelines to design widgets for interpretability visualization.

**Figure 9:**
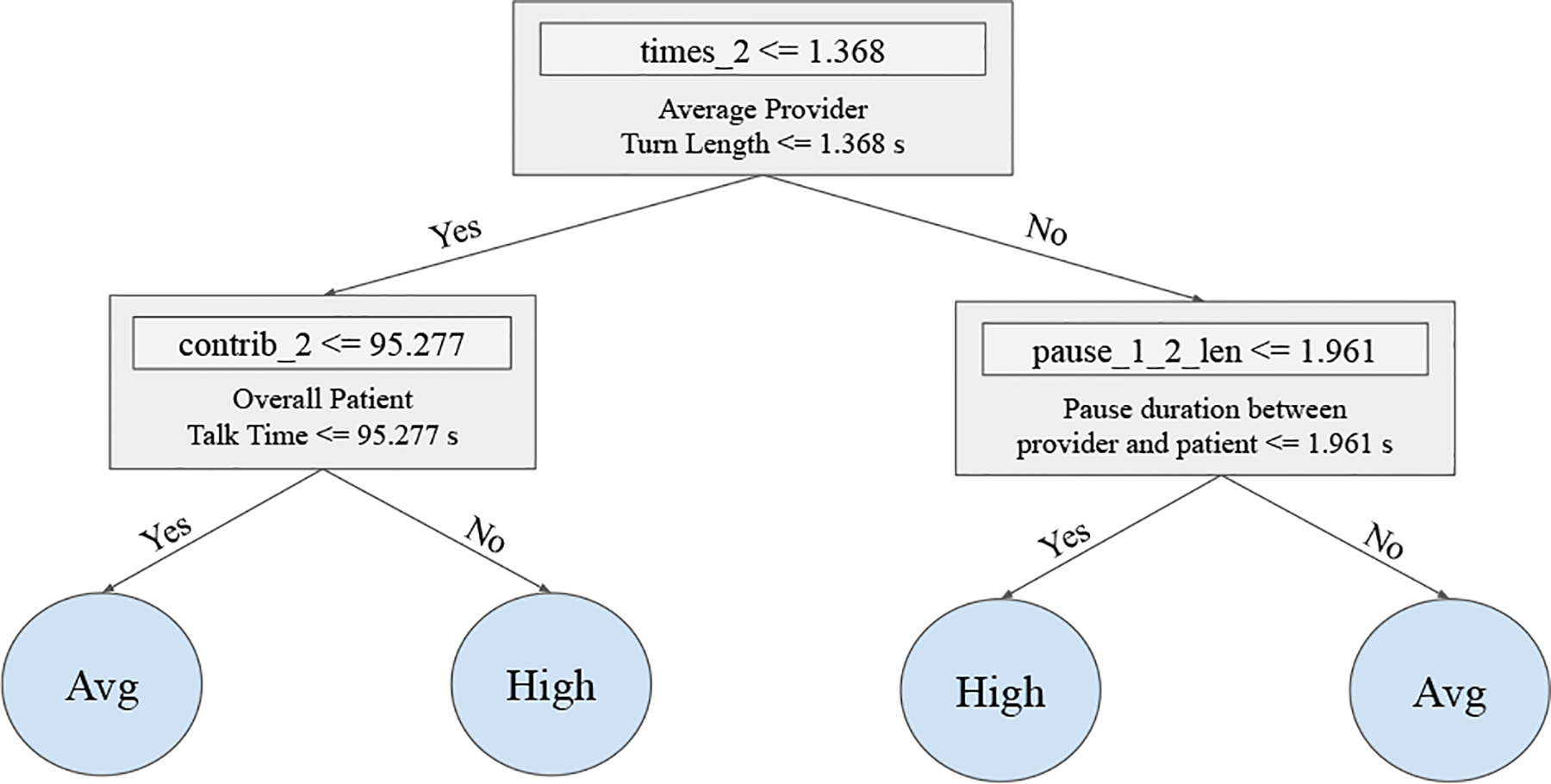
An interpretation of an exemplar decision tree model for warmth. The model is interpretable in the sense that we exactly know which conditions will lead to which outcome and we can explain the decision for every individual slice

Given that we used globally interpretable models such as Decision Tree Classifiers or Logistic Regressions in our experiments, we are able to visualize decisions trees (see Fig. 9) and easily give providers a straight-forward way to understand how the different nonverbal features of the underlining model interplay to determine the final prediction (magnitude of a given social signal). Similarly, regression weights can also be used as absolute indicators of feature trends.

To increase engagement with our interpretable models, we propose to add a widget/modal attached to each data point in a plot to help providers understand the features in the model that are influencing that specific data point. We believe that adding hints to understand how specific features influence social signals will lead to better informed decisions when providers want to modify their communication behavior.

### 7.7 Limitations and Future Work

While our tool presents varied takeaways and implications for different aspects of communication feedback in patient-provider interactions, we also acknowledge several limitations. Our use of recorded patient visits offered real world data to train our SSP models, yet this data was sparse which led to challenges like imbalanced classes for social signals, due to which we had to reduce the granularity of our predictions. Our data was unimodal (audio-only) and hence more prominent gestures (e.g., head movements, gaze, etc.) that can be traced through other modalities was not yet integrated. Our modeling methods were also constrained to classical machine learning approaches to account for smaller dataset and easier interpretability. Despite these challenges, we were able to model four out of twelve RIAS social signals to date. Future work could also explore privacy aware sensing [6, 65, 94] models.

Further, the distribution of patients and providers in our dataset was imbalanced both in terms of behaviors and demographics. We are in the process of collecting more data to bridge this gap. While our user study was preliminary with a small sample, we used a rigorous mixed methods study design that generated a depth of informative insights and structured protocol that can be expanded in future research. Our work also explored an individual form of feedback through social signals and future work will explore its effect along side other prior-explored aspects to get a quantified measure of impact.

Now that the utility of of ConverSense and our approach more broadly has been benchmarked, our future work will focus on enhancing the system in preparation for a contextual evaluation in real clinical settings to demonstrate the system’s impact. Enhancements will include the collection of additional data to improve the pipeline and refining the ConverSense interface, which could be informed by feedback practices of communication coaches [2] and narrative storytelling approaches [68].

While this system is an initial attempt towards understanding provider behaviors, researchers should be aware of the potential negative consequences if this sensitive information is used in a wrong way. Potential misuse could occur if these data is used by systems geared towards evaluating physician performance.

## 8 CONCLUSION

Social interactions form an integral part of patient-provider communication, and social signals have proven to be potential indicators to measure different dimensions of the quality of that communication. In this work, we explored how non-verbal vocal behaviors (i.e., vocalics) can be used to computationally track social signals such as dominance, interactiveness, engagement, and warmth. Upon successfully developing a fully interpretable machine learning model, we introduce *ConverSense* a web-based tool to help providers visualize their social interactions with patients. We conducted a pilot user-study with five providers, each interacting with two patient actors to explore their perception of social signals and data-driven models as instruments for communication feedback.

In our work we found that social signals can be used to identify moments of change in affect, but that social signals alone are not sufficient to provide actionable communication feedback to providers. We also learned that people interpret social signals differently, and this misalignment can reduce trust in the automatic assessment provided by ConverSense.

Our findings pushed us to consider contextual and actionable feedback as a core element that is needed to enable providers to act on system-provided feedback, and we discussed strategies for future work that can enhance the usability and utility of this approach. Our hope is that tools informed by these findings will ultimately help healthcare providers improve their communication with all patients, raise awareness of potential implicit bias in that communication, and ultimately improve the quality and equity of healthcare.

## Figures and Tables

**Table 1:** Data Clustering coding used for classification across the four social signals at the basis of our analysis. The 1–6 indexes show how the scoring is distributed across the low/baseline/high levels. In parenthesis the number of data points available at that level. We discard all low labels due to absence of meaningful number of samples.

Signal	Low (1–2)	Neutral (3)	High (4–6)
Provider Dominance	x	162	369
Patient Dominance	7	480	44
Provider Interactiveness	x	403	128
Patient Interactiveness	x	397	134
Provider Warmth	4	351	176
Patient Warmth	x	367	164
Provider Engagement	x	26	505
Patient Engagement	1	42	488

**Table 2:** Results showing comparison of accuracy and macro-F1 scores across different interpretable models’ performance averaged over providers for the *neutral*-*high* classification. LR, DTC, SVM, RF and GBDT stand for Logistic Regression, Decision Tree Classifier, Support Vector Machine, Random Forest and Gradient Boosted Decision Trees respectively. Despite the small sample size and class imbalance, most models perform better than a macro-F1 threshold of 0.5

Social Signal	Metric	LR	DTC	SVM (Linear)	SVM (Radial)	RF	GBDT
Provider Dominance	Accuracy	**0.719**	0.673	0.703	0.704	0.722	0.717
	F1 Score	**0.658**	0.606	0.650	0.614	0.651	0.657
Provider Interactiveness	Accuracy	**0.660**	0.641	0.657	0.690	0.692	0.616
	F1 Score	**0.559**	0.550	0.548	0.519	0.523	0.525
Provider Engagement	Accuracy	0.863	**0.947**	0.852	0.903	0.898	0.934
	F1 Score	0.547	**0.736**	0.566	0.605	0.651	0.672
Provider Warmth	F1 Score	0.556	0.543	**0.581**	0.581	0.571	0.565
	F1 Score	0.515	0.501	**0.544**	0.460	0.521	0.515
Patient Dominance	Accuracy	**0.975**	0.968	0.973	0.973	0.961	0.970
	F1 Score	**0.910**	0.841	0.892	0.893	0.840	0.842
Patient Interactiveness	Accuracy	**0.651**	0.641	0.660	0.679	0.647	0.653
	F1 Score	**0.559**	0.509	0.557	0.525	0.523	0.519
Patient Engagement	Accuracy	0.826	0.663	0.827	**0.883**	0.804	0.826
	F1 Score	0.556	0.529	0.551	**0.599**	0.557	0.594
Patient Warmth	Accuracy	0.517	0.544	0.551	0.634	**0.610**	0.605
	F1 Score	0.537	0.562	0.568	0.604	**0.614**	0.603

**Table 3:** Definitions and expected behaviors of selected RIAS signals in patient-provider interactions

Name	Description	Applies to	Desired Behavior
Dominance	Confidently aggressive and self-assured; but can be domineering at times	Patient	High
		Provider	Low
Interactiveness	Keeping oneself involved in the conversation	Patient	High
		Provider	High
Engagement	Being receptive and reacting appropriately to others	Patient	High
		Provider	High
Warmth	Showing kindness and caring	Patient	High
		Provider	High

**Table 4:** Summary of participants in the user study

ID	Age	Gender	Race	Medical Education	Healthcare Experience
P1	47	Woman	White	MD	21 years
P2	36	Man	White	MD	4 years
P3	58	Man	White	MD	32 years
P4	36	Man	White	MD	8 years
P5	53	Man	East Asian	MD	25 years

## CCS CONCEPTS

• **Human-centered computing → Ubiquitous and mobile computing systems and tools**.

## Footnotes

1 Our interdisciplinary team includes a practicing clinician, who is also one of the senior authors of this paper. All domain-centered considerations and interface redesigns were continuously assessed through their perspective throughout the project.

2
https://flask.palletsprojects.com/en/2.3.x/

3
https://plotly.com/

4
https://www.nei.nih.gov/learn-about-eye-health/eye-conditions-and-diseases/color-blindness/types-color-vision-deficiency

## Appendix A FEATURE LIST

We use the features below for feature engineering the SSP pipeline.

1. *Turn-Taking Behavior* — mean, std of average turn length, number of turns, contribution % of slice [2 × 3 = 6]
2. *Interruptions* — mean, std of number of interruptions and turn length after interruption for patient-provider and provider-patient turn change [2 × 2 × 2 = 8]
3. *Pauses* — mean, std of number of pauses (), pause duration between turn change (2) and turn kept (2) [2 × 2 + 4 × 2 = 12]
4. *Mel Frequency Cepstral Coefficients* — min, max, mean, std of 13 features for two speakers [13 × 4 × 2 = 104]
5. *Spectral Centroid* — min, max, mean, std for 2 speakers [2 × 4 = 8]
6. *Spectral Rolloff* — min, max, mean, std for 2 speakers [2 × 4 = 8]
7. *Pitch* — min, max, mean, std for 2 speakers [2 × 4 = 8]
8. *Root Mean Square Energy* — min, max, mean, std for 2 speakers [2 × 4 = 8]
9. *Zero Crossing Rate* — min, max, mean, std for 2 speakers [2 × 4 = 8]
10. *Intensity* — min, max, mean, std for 2 speakers [2 × 4 = 8]

This results in a total of 178 features that are used in the ML models in Section 3

## Appendix B PATIENT ACTOR SCRIPTS

The below scripts are used by patient actors to ensure standardized patient behavior

### B.1 SCENARIO 1. 25–50 YEAR-OLD BLACK OR WHITE PATIENT WITH DIABETES

1. **Opening Statement:** My diabetes is getting worse. My last doctor told me it’s because I’m lazy and don’t take care of myself, and I need more guidance than that.
2. **If provider questions you about why you feel this way, respond with:** “I’m really hoping to walk out of here with some real advice today for my diabetes. Sometimes I just feel like the whole system is against us. When talking with medical professionals, I’ve never felt that I’ve been treated as a reasonable person. It seems like they treat me differently because of the way I look.”
3. **History of Present Illness:** You are a 45–65-year-old patient who comes in with chief complaint of worsening diabetes. Your blood sugar has been mildly elevated and your last doctor prescribed metformin, but you want to avoid the need for additional medications or insulin. You have been pretty good at taking the metformin doses (occasional missed dose, but you take it most days as prescribed). You’re here today to learn more about what changes you can make to your diet and lifestyle to help control your blood sugar.
4. **Past Medical History:** You were diagnosed 4 years ago with Type II diabetes and metabolic syndrome (increased blood pressure, abnormal cholesterol, and excess body weight). You believe that diabetes is “just a part of life”. Your previous doctor told you that you were lazy, and don’t take care of yourself 6 months ago, at your last visit. You can add: “She made me feel hopeless, treated me like it was all my fault, didn’t give me anything to do. Really made me feel bad about myself. I know I need to get into better shape, but I am not lazy. I have a job, but people make assumptions when they see me.”
5. **Exercise Routine:** You don’t exercise regularly. Some weeks you walk for 10 to 20 minutes a couple times and other weeks you don’t.
6. **Diet:** Your diet consists primarily of fast food or food from the convenience store; pizza, microwavable meals, and snack foods, like donuts and cookies, stuff you can eat quickly on the way to or from work. You prefer soda, coffee, and energy drinks. You currently experience fatigue, and caffeine helps to stay motivated during the day. You don’t like to cook unless it’s for a large family gathering.
7. **Job:** You work as an elementary school teacher (4th grade).
8. **Medications:** You take Metformin 1000mg twice a day.
9. **When do you measure blood sugar?** You take it here and there, but no one has shown you how to do it.
10. **Drugs/Alcohol:** When you were younger you did “all sorts of stuff” like “you know, the usual, alcohol and some other stuff”, but you quit 20 years ago. Sometimes I still smoke marijuana and drink a couple of beers.
11. **Associated Signs and Symptoms** If asked about other things, like lab work, medication, diet/lifestyle: “I don’t know, maybe sometimes, not really sure…” Ok to acknowledge you feel fatigued many days.
12. **Family History:** Both mother and grandmother were diagnosed with Type II diabetes in their mid-40s.
13. **Social history:** You are divorced and live by yourself in a small apartment near the school where you work. You do not get along with your ex. You see your two kids once a week and pay child support.
14. **General:** You struggled to get workers compensation from your employer and can’t afford to take the time off work or pay for another doctor’s visit. You fear being able to keep up with work. You have been living in low-income housing and don’t know how the family would get by if you lost your job.

### B.2 SCENARIO 2. 25–50 YEAR-OLD BLACK OR WHITE PATIENT WITH CHRONIC PAIN (denied meds)

1. **Opening Statement:** I went to the ER two weeks ago, and they wouldn’t give me any meds. I feel like they weren’t even treating me like a human being – they were treating me like a druggie.
2. **If provider questions you about why you feel this way, respond with:** “I’m really hoping to walk out of here with some real help today for my pain. Our whole system is against us. I’ve never felt that I’ve been treated as a reasonable person when talking to medical professionals. It seems like they treat me differently because of the way I look.
3. **History of Present Illness** You are a 25–50 year-old patient with a chief complaint of chronic pain. You describe the pain as aching and sometimes burning all over your back and right shoulder. The pain has worsened in the past month and the situation with your employer has left you feeling hopeless and fatigued. Overall, you appear very tired.
4. **Past Medical History** You began experiencing chronic pain in your back and right shoulder after a work accident that occurred over a year ago. A beam fell and hit your shoulder. You went into a physician after the accident and were given pain medication for three months, but you don’t remember the name of it. Since then, you have not been back.
5. **Exercise routine** You don’t exercise regularly, but your job is physically demanding.
6. **Diet** You have good eating habits. You avoid processed food and eating out for the most part. You cook most of your food yourself.
7. **Job** You are a construction worker.
8. **Medication** Two weeks ago, your pain was so bad you went to the ER, and they wouldn’t give you meds. You have tried ibuprofen and acetaminophen but didn’t find they made much of a difference.
  “I need something stronger. The pain is awful and makes it hard for me to work. Being on the job all day is hard enough but with this shoulder, it’s almost impossible. I need something strong for a while, until it gets better”
9. **Drugs/Alcohol** To cope with the pain, you have begun to drink a lot in the evenings, (usually 1–2 beers per night but 3–4 a day when pain is bad) to numb the pain. You express that alcohol takes a bit of the edge off but is not always strong enough to mask the pain. You are curious if you can get something stronger so that you can continue working and not have to continue paying for doctors’ visits. If you had to rate the effectiveness of the pain suppressants you have used, the pain medication was the most effective, then alcohol.
10. **Associated Signs and Symptoms:** When asked about sleep you say you often wake during the night and are unable to go back to sleep either from pain or worry. You would rate the pain currently as an 8/10 and that at its worst it is 10/10. Otherwise, healthy overall. If asked about other things: “I don’t know, maybe sometimes, not really sure…”
11. **Social History:** You are divorced and live by yourself in a small apartment near the construction site where you work. You do not get along with your ex. You see your two kids once a week and pay child support.
12. **General:** You struggled to get workers compensation from your employer and can’t afford to take the time off work or pay for another doctor’s visit. You fear losing your job since the accident and are worried what effect that would have on your children. You have been living in low-income housing and don’t know how the family would get by if you lost your job.

## References

[1] Arakawa Riku and Yakura Hiromu. 2019. REsCUE: A framework for REal-time feedback on behavioral CUEs using multimodal anomaly detection. In Proceedings of the 2019 CHI Conference on Human Factors in Computing Systems. 1–13.

[2] Arakawa Riku and Yakura Hiromu. 2020. INWARD: A Computer-Supported Tool for Video-Reflection Improves Efficiency and Effectiveness in Executive Coaching. In Proceedings of the 2020 CHI Conference on Human Factors in Computing Systems. 1–13.

[3] Bartlett Gillian, Blais Régis, Tamblyn Robyn, Clermont Richard J, and MacGibbon Brenda. 2008. Impact of patient communication problems on the risk of preventable adverse events in acute care settings. Cmaj 178, 12 (2008), 1555–1562. 18519903 10.1503/cmaj.070690 PMC2396356

[4] Bascom Emily, Reggie Casanova-Perez Kelly Tobar, Manas Satish Bedmutha Harshini Ramaswamy, Pratt Wanda, Sabin Janice, Wood Brian, Weibel Nadir, and Hartzler Andrea. 2024. Designing Communication Feedback Systems To Reduce Healthcare Providers’ Implicit Biases In Patient Encounters. In Proceedings of the 2024 CHI Conference on Human Factors in Computing Systems. 10.1145/3613904.3642756 PMC11204363 38933286

[5] Bedmutha Manas Satish, Bascom Emily, Reggie Casanova-Perez, Tobar Kelly, Sabin Janice, Pratt Wanda, Wood Brian, Hartzler Andrea, and Weibel Nadir. 2023. Towards Designing Visualizations to Understand Social Signals in Patient-Provider Communication. (2023).

[6] Bedmutha Manas Satish, Bedmutha Poorva Satish, and Weibel Nadir. 2023. Privacy-Aware Respiratory Symptom Detection in-the-wild. In Adjunct Proceedings of the 2023 ACM International Joint Conference on Pervasive and Ubiquitous Computing & the 2023 ACM International Symposium on Wearable Computing. Association for Computing Machinery, New York, NY, USA, 428–432. 10.1145/3594739.3610733

[7] Bedmutha Manas Satish, Bhat Amrit, Mangal Sabrina, Bascom Emily, Pratt Wanda, Wood Brian, Sabin Janice, Weibel Nadir, and Hartzler Andrea. 2023. Towards inferring implicit bias in clinical interactions using social signals. AMIA Annual Symposium. AI Showcase Stage III (2023).

[8] Beyan Cigdem, Katsageorgiou Vasiliki-Maria, and Murino Vittorio. 2017. Moving as a leader: Detecting emergent leadership in small groups using body pose. In Proceedings of the 25th ACM international conference on Multimedia. 1425–1433.

[9] Borrie Stephanie A, Barrett Tyson S, Liss Julie M, and Berisha Visar. 2020. Sync pending: Characterizing conversational entrainment in dysarthria using a multidimensional, clinically informed approach. Journal of Speech, Language, and Hearing Research 63, 1 (2020), 83–94. 10.1044/2019_JSLHR-19-00194 PMC7213480 31855608

[10] Borrie Stephanie A, Barrett Tyson S, Willi Megan M, and Berisha Visar. 2019. Syncing up for a good conversation: A clinically meaningful methodology for capturing conversational entrainment in the speech domain. Journal of Speech, Language, and Hearing Research 62, 2 (2019), 283–296. 10.1044/2018_JSLHR-S-18-0210 PMC6436892 30950701

[11] Brooke John. 1996. Sus: a “quick and dirty’usability. Usability evaluation in industry 189, 3 (1996), 189–194.

[12] Buller Mary Klein and Buller David B. 1987. Physicians’ communication style and patient satisfaction. Journal of health and social behavior (1987), 375–388. 3429807

[13] Burgess Eleanor R, Jankovic Ivana, Austin Melissa, Cai Nancy, Kapuścińska Adela, Currie Suzanne, Overhage J Marc, Poole Erika S, and Kaye Jofish. 2023. Healthcare AI Treatment Decision Support: Design Principles to Enhance Clinician Adoption and Trust. In Proceedings of the 2023 CHI Conference on Human Factors in Computing Systems. 1–19.

[14] Burgess Eleanor R, Kaziunas Elizabeth, and Jacobs Maia. 2022. Care Frictions: A Critical Reframing of Patient Noncompliance in Health Technology Design. Proceedings of the ACM on Human-Computer Interaction 6, CSCW2 (2022), 1–31.37360538

[15] Burgoon Judee K, Floyd Kory, and Guerrero Laura K. 2010. Nonverbal communication theories of interpersonal adaptation. In The new SAGE handbook of communication science. Sage, 93–110.

[16] Burgoon Judee K, Magnenat-Thalmann Nadia, Pantic Maja, and Vinciarelli Alessandro. 2017. Social signal processing. Cambridge University Press.

[17] Bussone Adrian, Stumpf Simone, and O’Sullivan Dympna. 2015. The role of explanations on trust and reliance in clinical decision support systems. In 2015 international conference on healthcare informatics. IEEE, 160–169.

[18] Cadsby C Bram, Song Fei, Engle-Warnick Jim, and Fang Tony. 2019. Invoking social comparison to improve performance by ranking employees: The moderating effects of public ranking, rank pay, and individual risk attitude. Journal of Economic Psychology 72 (2019), 64–79.

[19] Campbell Donald J and Furrer David M. 1995. Goal setting and competition as determinants of task performance. Journal of Organizational Behavior 16, 4 (1995), 377–389.

[20] Casanova-Perez Reggie, Apodaca Calvin, Bascom Emily, Mohanraj Deepthi, Lane Cezanne, Vidyarthi Drishti, Beneteau Erin, Sabin Janice, Pratt Wanda, Weibel Nadir, 2021. Broken down by bias: Healthcare biases experienced by BIPOC and LGBTQ+ patients. In AMIA Annual Symposium Proceedings, Vol. 2021. American Medical Informatics Association, 275. 35308990 PMC8861755

[21] Cegala Donald J and Broz Stefne Lenzmeier. 2002. Physician communication skills training: a review of theoretical backgrounds, objectives and skills. Medical education 36, 11 (2002), 1004–1016. 12406260 10.1046/j.1365-2923.2002.01331.x

[22] Chatzimparmpas Angelos, Martins Rafael Messias, Jusufi Ilir, Kucher Kostiantyn, Rossi Fabrice, and Kerren Andreas. 2020. The state of the art in enhancing trust in machine learning models with the use of visualizations. In Computer Graphics Forum, Vol. 39. Wiley Online Library, 713–756.

[23] Chawla Nitesh V, Bowyer Kevin W, Hall Lawrence O, and Kegelmeyer W Philip. 2002. SMOTE: synthetic minority over-sampling technique. Journal of artificial intelligence research 16 (2002), 321–357.

[24] Chen Zhi-Hong. 2014. Learning preferences and motivation of different ability students for social-competition or self-competition. Journal of Educational Technology & Society 17, 1 (2014), 283–293.

[25] Collins Lauren G, Schrimmer Anne, Diamond James, and Burke Janice. 2011. Evaluating verbal and non-verbal communication skills, in an ethnogeriatric OSCE. Patient education and counseling 83, 2 (2011), 158–162. 20561763 10.1016/j.pec.2010.05.012

[26] Cooper Lisa A, Roter Debra L, Carson Kathryn A, Beach Mary Catherine, Sabin Janice A, Greenwald Anthony G, and Inui Thomas S. 2012. The associations of clinicians’ implicit attitudes about race with medical visit communication and patient ratings of interpersonal care. American journal of public health 102, 5 (2012), 979–987. 22420787 10.2105/AJPH.2011.300558 PMC3483913

[27] Coria Juan M., Bredin Hervé, Ghannay Sahar, and Rosset Sophie. 2021. Overlap-Aware Low-Latency Online Speaker Diarization Based on End-to-End Local Segmentation. In 2021 IEEE Automatic Speech Recognition and Understanding Workshop (ASRU). 1139–1146.

[28] Cuddy Amy JC, Glick Peter, and Beninger Anna. 2011. The dynamics of warmth and competence judgments, and their outcomes in organizations. Research in organizational behavior 31 (2011), 73–98.

[29] Lascio Elena Di, Gashi Shkurta, and Santini Silvia. 2018. Unobtrusive assessment of students’ emotional engagement during lectures using electrodermal activity sensors. Proceedings of the ACM on Interactive, Mobile, Wearable and Ubiquitous Technologies 2, 3 (2018), 1–21.

[30] Diprose William K, Buist Nicholas, Hua Ning, Thurier Quentin, Shand George, and Robinson Reece. 2020. Physician understanding, explainability, and trust in a hypothetical machine learning risk calculator. Journal of the American Medical Informatics Association 27, 4 (2020), 592–600. 32106285 10.1093/jamia/ocz229 PMC7647292

[31] Dirks Lisa, Beneteau Erin, Sabin Janice, Pratt Wanda, Lane Cezanne, Bascom Emily, Casanova-Perez Reggie, Rizvi Naba, Weibel Nadir, and Hartzler Andrea. 2022. Battling Bias in Primary Care Encounters: Informatics Designs to Support Clinicians. In CHI Conference on Human Factors in Computing Systems Extended Abstracts. 1–9. 10.1145/3491101.3519825 PMC9128862 35615338

[32] Ericsson K Anders and Simon Herbert A. 1980. Verbal reports as data. Psychological review 87, 3 (1980), 215.

[33] Faucett Heather A, Lee Matthew L, and Carter Scott. 2017. I should listen more: real-time sensing and feedback of non-verbal communication in video telehealth. Proceedings of the ACM on Human-Computer Interaction 1, CSCW (2017), 1–19.

[34] FitzGerald Chloë and Hurst Samia. 2017. Implicit bias in healthcare professionals: a systematic review. BMC medical ethics 18, 1 (2017), 1–18. 28249596 10.1186/s12910-017-0179-8 PMC5333436

[35] Fouse Adam, Weibel Nadir, Hutchins Edwin, and Hollan James D. 2011. ChronoViz: a system for supporting navigation of time-coded data. In CHI’11 Extended Abstracts on Human Factors in Computing Systems. 299–304.

[36] Friard Olivier and Gamba Marco. 2016. BORIS: a free, versatile open-source event-logging software for video/audio coding and live observations. Methods in ecology and evolution 7, 11 (2016), 1325–1330.

[37] Gashi Shkurta, Lascio Elena Di, and Santini Silvia. 2019. Using unobtrusive wearable sensors to measure the physiological synchrony between presenters and audience members. Proceedings of the ACM on Interactive, Mobile, Wearable and Ubiquitous Technologies 3, 1 (2019), 1–19.34164595

[38] Gossman Marion and Miller Judi H. 2012. ‘The third person in the room’: Recording the counselling interview for the purpose of counsellor training–barrier to relationship building or effective tool for professional development? Counselling and Psychotherapy Research 12, 1 (2012), 25–34.

[39] Green Alexander R, Carney Dana R, Pallin Daniel J, Ngo Long H, Raymond Kristal L, Iezzoni Lisa I, and Banaji Mahzarin R. 2007. Implicit bias among physicians and its prediction of thrombolysis decisions for black and white patients. Journal of general internal medicine 22, 9 (2007), 1231–1238. 17594129 10.1007/s11606-007-0258-5 PMC2219763

[40] Greene Jessica, Hibbard Judith H, Alvarez Carmen, and Overton Valerie. 2016. Supporting patient behavior change: approaches used by primary care clinicians whose patients have an increase in activation levels. The Annals of Family Medicine 14, 2 (2016), 148–154. 26951590 10.1370/afm.1904 PMC4781518

[41] Hagiwara Nao, Lafata Jennifer Elston, Mezuk Briana, Vrana Scott R, and Fetters Michael D. 2019. Detecting implicit racial bias in provider communication behaviors to reduce disparities in healthcare: challenges, solutions, and future directions for provider communication training. Patient education and counseling 102, 9 (2019), 1738–1743. 31036330 10.1016/j.pec.2019.04.023 PMC7269129

[42] Hall Judith A, Roter Debra L, and Katz Nancy R. 1988. Meta-analysis of correlates of provider behavior in medical encounters. Medical care 26, 7 (1988), 657–675. 3292851 10.1097/00005650-198807000-00002

[43] Hall William J, Chapman Mimi V, Lee Kent M, Merino Yesenia M, Thomas Tainayah W, Payne B Keith, Eng Eugenia, Day Steven H, and Coyne-Beasley Tamera. 2015. Implicit racial/ethnic bias among health care professionals and its influence on health care outcomes: a systematic review. American journal of public health 105, 12 (2015), e60–e76. 10.2105/AJPH.2015.302903 PMC4638275 26469668

[44] Hart Sandra G and Staveland Lowell E. 1988. Development of NASA-TLX (Task Load Index): Results of empirical and theoretical research. In Advances in psychology. Vol. 52. Elsevier, 139–183.

[45] Hartzler AL, Patel RA, Czerwinski M, Pratt W, Roseway A, Chandrasekaran N, and Back A. 2014. Real-time feedback on nonverbal clinical communication. Methods of information in medicine 53, 05 (2014), 389–405. 24970354 10.3414/ME13-02-0033

[46] Henry Stephen G, Fuhrel-Forbis Andrea, Rogers Mary AM, and Eggly Susan. 2012. Association between nonverbal communication during clinical interactions and outcomes: a systematic review and meta-analysis. Patient education and counseling 86, 3 (2012), 297–315. 21824738 10.1016/j.pec.2011.07.006

[47] Hoffman Kelly M, Trawalter Sophie, Axt Jordan R, and Oliver M Norman. 2016. Racial bias in pain assessment and treatment recommendations, and false beliefs about biological differences between blacks and whites. Proceedings of the National Academy of Sciences 113, 16 (2016), 4296–4301. 10.1073/pnas.1516047113 PMC4843483 27044069

[48] Chiao-yin Hsiao Joey, Jih Wan-rong, and Yung-jen Hsu Jane. 2012. Recognizing continuous social engagement level in dyadic conversation by using turn-taking and speech emotion patterns. In Workshops at the Twenty-Sixth AAAI Conference on Artificial Intelligence.

[49] Hung Hayley, Jayagopi Dinesh, Yeo Chuohao, Friedland Gerald, Ba Sileye, Odobez Jean-Marc, Ramchandran Kannan, Mirghafori Nikki, and Gatica-Perez Daniel. 2007. Using audio and video features to classify the most dominant person in a group meeting. In Proceedings of the 15th ACM international conference on Multimedia. 835–838.

[50] Jayagopi Dinesh Babu, Hung Hayley, Yeo Chuohao, and Gatica-Perez Daniel. 2009. Modeling dominance in group conversations using nonverbal activity cues. IEEE Transactions on Audio, Speech, and Language Processing 17, 3 (2009), 501–513.

[51] Joo Hanbyul, Simon Tomas, Cikara Mina, and Sheikh Yaser. 2019. Towards social artificial intelligence: Nonverbal social signal prediction in a triadic interaction. In Proceedings of the IEEE/CVF Conference on Computer Vision and Pattern Recognition. 10873–10883.

[52] Kanter Jonathan W, Rosen Daniel C, Manbeck Katherine E, Branstetter Heather ML, Kuczynski Adam M, Corey Mariah D, Maitland Daniel WM, and Williams Monnica T. 2020. Addressing microaggressions in racially charged patient-provider interactions: a pilot randomized trial. BMC Medical Education 20 (2020), 1–14. 10.1186/s12909-020-02004-9 PMC7092438 32209082

[53] Kraft-Todd Gordon T, Reinero Diego A, Kelley John M, Heberlein Andrea S, Baer Lee, and Riess Helen. 2017. Empathic nonverbal behavior increases ratings of both warmth and competence in a medical context. PloS one 12, 5 (2017), e0177758. 28505180 10.1371/journal.pone.0177758 PMC5432110

[54] Kron Frederick W, Fetters Michael D, Scerbo Mark W, White Casey B, Lypson Monica L, Padilla Miguel A, Gliva-McConvey Gayle A, Belfore Lee A II, West Temple, Wallace Amelia M, 2017. Using a computer simulation for teaching communication skills: A blinded multisite mixed methods randomized controlled trial. Patient education and counseling 100, 4 (2017), 748–759. 27939846 10.1016/j.pec.2016.10.024 PMC5385273

[55] Landsberger Henry A. 1958. Hawthorne Revisited: Management and the Worker, Its Critics, and Developments in Human Relations in Industry. (1958).

[56] LaRosa Emily and Danks David. 2018. Impacts on trust of healthcare AI. In Proceedings of the 2018 AAAI/ACM Conference on AI, Ethics, and Society. 210–215.

[57] Liu Chunfeng, Lim Renee L, McCabe Kathryn L, Taylor Silas, Calvo Rafael A, 2016. A web-based telehealth training platform incorporating automated nonverbal behavior feedback for teaching communication skills to medical students: a randomized crossover study. Journal of medical Internet research 18, 9 (2016), e6299. 10.2196/jmir.6299 PMC5037316 27619564

[58] Liu Chunfeng, Scott Karen M, Lim Renee L, Taylor Silas, and Calvo Rafael A. 2016. EQClinic: a platform for learning communication skills in clinical consultations. Medical education online 21, 1 (2016), 31801. 27476537 10.3402/meo.v21.31801 PMC4967711

[59] Lyle John. 2003. Stimulated recall: A report on its use in naturalistic research. British educational research journal 29, 6 (2003), 861–878.

[60] Manusov Valerie Lynn. 2014. The sourcebook of nonverbal measures: Going beyond words. Psychology Press.

[61] Martinez EL. 2007. Patient-centered communication with vulnerable populations. Promising Practices for Addressing Health Literacy. Institute of Medicine–Roundtable on Health Literacy (2007).

[62] Mauksch Larry B, Hillenburg Lena, and Robins Lynne. 2001. The Establishing Focus protocol: Training for collaborative agenda setting and time management in the medical interview. Families, Systems, & Health 19, 2 (2001), 147.

[63] Mead Nicola and Bower Peter. 2000. Measuring patient-centredness: a comparison of three observation-based instruments. Patient education and counseling 39, 1 (2000), 71–80. 11013549 10.1016/s0738-3991(99)00092-0

[64] Mollo Vanina and Falzon Pierre. 2004. Auto-and allo-confrontation as tools for reflective activities. Applied ergonomics 35, 6 (2004), 531–540. 15374760 10.1016/j.apergo.2004.06.003

[65] Mollyn Vimal, Ahuja Karan, Verma Dhruv, Harrison Chris, and Goel Mayank. 2022. SAMoSA: Sensing Activities with Motion and Subsampled Audio. Proceedings of the ACM on Interactive, Mobile, Wearable and Ubiquitous Technologies 6, 3 (2022), 1–19.

[66] Montgomery Martin. 2013. An introduction to language and society. Routle (2013).

[67] Nelson Alan. 2002. Unequal treatment: confronting racial and ethnic disparities in health care. Journal of the national medical association 94, 8 (2002), 666. 12152921 PMC2594273

[68] Fernandez Nieto Gloria Milena, Kitto Kirsty, Shum Simon Buckingham, and Martinez-Maldonado Roberto. 2022. Beyond the learning analytics dashboard: Alternative ways to communicate student data insights combining visualisation, narrative and storytelling. In International Conference on Learning Analytics and Knowledge 2022. Association for Computing Machinery (ACM), 219–229.

[69] Palaniappan Kavitha and Noor Norah Md. 2022. Gamification strategy to support self-directed learning in an online learning environment. International Journal of Emerging Technologies in Learning (iJET) 17, 3 (2022), 104–116.

[70] Peek Monica E, Odoms-Young Angela, Quinn Michael T, Gorawara-Bhat Rita, Wilson Shannon C, and Chin Marshall H. 2010. Race and shared decision-making: perspectives of African-Americans with diabetes. Social science & medicine 71, 1 (2010), 1–9. 20409625 10.1016/j.socscimed.2010.03.014 PMC2885527

[71] Pentland Alex. 2010. Honest signals: how they shape our world. MIT press.

[72] Peters Rifca, Broekens Joost, and Neerincx Mark A. 2017. Robots educate in style: The effect of context and non-verbal behaviour on children’s perceptions of warmth and competence. In 2017 26th IEEE international symposium on robot and human interactive communication (RO-MAN). IEEE, 449–455.

[73] PlÖtz Thomas. 2021. Applying machine learning for sensor data analysis in interactive systems: Common pitfalls of pragmatic use and ways to avoid them. ACM Computing Surveys (CSUR) 54, 6 (2021), 1–25.

[74] Prabaswari Atyanti Dyah, Basumerda Chancard, and Utomo Bagus Wahyu. 2019. The mental workload analysis of staff in study program of private educational organization. In IOP Conference Series: Materials Science and Engineering, Vol. 528. IOP Publishing, 012018.

[75] Prochaska James O and DiClemente Charles C. 2005. The transtheoretical approach. Handbook of psychotherapy integration 2 (2005), 147–171.

[76] Ribeiro Marco Tulio, Singh Sameer, and Guestrin Carlos. 2016. “ Why should i trust you?” Explaining the predictions of any classifier. In Proceedings of the 22nd ACM SIGKDD international conference on knowledge discovery and data mining. 1135–1144.

[77] Rizvi Naba, Ramaswamy Harshini, Casanova-Perez Reggie, Hartzler Andrea, and Weibel Nadir. 2022. Making Hidden Bias Visible: Designing a Feedback Ecosystem for Primary Care Providers. arXiv preprint arXiv:2204.07897 (2022).

[78] Roter Debra and Larson Susan. 2002. The Roter interaction analysis system (RIAS): utility and flexibility for analysis of medical interactions. Patient education and counseling 46, 4 (2002), 243–251. 11932123 10.1016/s0738-3991(02)00012-5

[79] Roter Debra L, Hall Judith A, Blanch-Hartigan Danielle, Larson Susan, and Frankel Richard M. 2011. Slicing it thin: new methods for brief sampling analysis using RIAS-coded medical dialogue. Patient education and counseling 82, 3 (2011), 410–419. 21239135 10.1016/j.pec.2010.11.019

[80] Roter Debra L, Larson Susan M, Beach Mary Catherine, and Cooper Lisa A. 2008. Interactive and evaluative correlates of dialogue sequence: a simulation study applying the RIAS to turn taking structures. Patient education and counseling 71, 1 (2008), 26–33. 18093788 10.1016/j.pec.2007.10.019 PMC2760431

[81] Rubenstein Lisa V, Mittman Brian S, Yano Elizabeth M, and Mulrow Cynthia D. 2000. From understanding health care provider behavior to improving health care: the QUERI framework for quality improvement. Medical care (2000), I129–I141. 10843277

[82] Sabin Janice A, Riskind Rachel G, and Nosek Brian A. 2015. Health care providers’ implicit and explicit attitudes toward lesbian women and gay men. American journal of public health 105, 9 (2015), 1831–1841. 26180976 10.2105/AJPH.2015.302631 PMC4539817

[83] Samrose Samiha, Daniel McDuff Robert Sim, Suh Jina, Rowan Kael, Hernandez Javier, Rintel Sean, Moynihan Kevin, and Czerwinski Mary. 2021. Meeting-Coach: An Intelligent Dashboard for Supporting Effective & Inclusive Meetings. In Proceedings of the 2021 CHI Conference on Human Factors in Computing Systems (<conf-loc>, Yokohama, <country>Japan</country>, </conf-loc>) (CHI ‘21). Association for Computing Machinery, New York, NY, USA, Article 252, 13 pages. 10.1145/3411764.3445615

[84] Scherer Stefan, Weibel Nadir, Morency Louis-Philippe, and Oviatt Sharon. 2012. Multimodal prediction of expertise and leadership in learning groups. In Proceedings of the 1st International Workshop on Multimodal Learning Analytics. 1–8.

[85] Schmitt Markus. 2022. Interpretable Machine Learning: Why and how to make your machine learning models interpretable. http://datarevenue.com (2022).

[86] Seo Woosuk, Buyuktur Ayse G, Verma Sanya, Kim Hyeryoung, Choi Sung Won, Sedig Laura, and Park Sun Young. 2021. Learning from Healthcare Providers’ Strategies: Designing Technology to Support Effective Child Patient-Provider Communication. In Proceedings of the 2021 CHI Conference on Human Factors in Computing Systems. 1–15.

[87] Shen Megan Johnson, Peterson Emily B, Costas-Muñiz Rosario, Hernandez Migda Hunter, Jewell Sarah T, Matsoukas Konstantina, and Bylund Carma L. 2018. The effects of race and racial concordance on patient-physician communication: a systematic review of the literature. Journal of racial and ethnic health disparities 5, 1 (2018), 117–140. 28275996 10.1007/s40615-017-0350-4 PMC5591056

[88] Sittikariyakul Pat, Jaturapatporn Darin, and Kirshen AJ. 2015. Acting as standardized patients enhances family medicine residents’ self-reported skills in palliative care. Advances in Health Sciences Education 20, 3 (2015), 645–654. 25256636 10.1007/s10459-014-9552-2

[89] Sladek Kimberly, Andreiu Alexandra, Rick Steven R., Schairer Cynthia E., Pratt Wanda, Sabin Janice A., Hartzler Andrea L., and Weibel Nadir. 2022. Using the Roter Interaction Analysis Scale (RIAS) with Positive and Negative Affect Scores to Signal Pivotal Moments in Patient-Provider Interactions. In AMIA Annual Symposium 2022. American Medical Informatics Association.

[90] Smiderle Rodrigo, Marques Leonardo, Coelho Jorge Artur P de M, Rigo Sandro J, and Jaques Patricia A. 2019. Studying the impact of gamification on learning and engagement of introverted and extroverted students. In 2019 IEEE 19th International Conference on Advanced Learning Technologies (ICALT), Vol. 2161. IEEE, 71–75.

[91] Street Richard L and Buller David B. 1987. Nonverbal response patterns in physician-patient interactions: A functional analysis. Journal of nonverbal behavior 11, 4 (1987), 234–253.

[92] Street Richard L Jr, Gordon Howard, and Haidet Paul. 2007. Physicians’ communication and perceptions of patients: is it how they look, how they talk, or is it just the doctor? Social science & medicine 65, 3 (2007), 586–598. 17462801 10.1016/j.socscimed.2007.03.036 PMC2811428

[93] Street Richard L Jr, Makoul Gregory, Arora Neeraj K, and Epstein Ronald M. 2009. How does communication heal? Pathways linking clinician–patient communication to health outcomes. Patient education and counseling 74, 3 (2009), 295–301. 19150199 10.1016/j.pec.2008.11.015

[94] Sun Ke, Chen Chen, and Zhang Xinyu. 2020. “Alexa, stop spying on me!” speech privacy protection against voice assistants. In Proceedings of the 18th conference on embedded networked sensor systems. 298–311.

[95] Truong Khiet P and van Leeuwen David A. 2005. Automatic detection of laughter. In Ninth European Conference on Speech Communication and Technology.

[96] Vinciarelli Alessandro, Pantic Maja, and Bourlard Hervé. 2009. Social signal processing: Survey of an emerging domain. Image and vision computing 27, 12 (2009), 1743–1759.

[97] Vogt Thurid, André Elisabeth, and Bee Nikolaus. 2008. EmoVoice—A framework for online recognition of emotions from voice. In Perception in Multimodal Dialogue Systems: 4th IEEE Tutorial and Research Workshop on Perception and Interactive Technologies for Speech-Based Systems, PIT 2008, Kloster Irsee, Germany, June 16–18, 2008. Proceedings 4. Springer, 188–199.

[98] Weibel Nadir, Rick Steven, Emmenegger Colleen, Ashfaq Shazia, Calvitti Alan, and Agha Zia. 2015. LAB-IN-A-BOX: semi-automatic tracking of activity in the medical office. Personal and Ubiquitous Computing 19, 2 (2015), 317–334.

[99] Williams Kristine, Herman Ruth, and Bontempo Daniel. 2013. Comparing audio and video data for rating communication. Western journal of nursing research 35, 8 (2013), 1060–1073. 23579475 10.1177/0193945913484813 PMC3729744

[100] Yu Chen, Aoki Paul M, and Woodruff Allison. 2004. Detecting user engagement in everyday conversations. arXiv preprint cs/0410027 (2004).

[101] Zheng Kai, Hanauer David A, Weibel Nadir, and Agha Zia. 2015. Computational ethnography: automated and unobtrusive means for collecting data in situ for human–computer interaction evaluation studies. Cognitive informatics for biomedicine: Human computer interaction in healthcare (2015), 111–140.

